# Associations Between Imaging‐Detected Pulp Calcification and Systemic Diseases: A Systematic Review, Thematic Analysis, and Meta‐Analysis

**DOI:** 10.1111/iej.70230

**Published:** 2026-07-19

**Authors:** José Evando da Silva‐Filho, Paula Maria da Costa Torres, Eduardo Diogo Gurgel‐Filho

**Affiliations:** ^1^ Department of Dental Radiology and Imaging, Faculty of Dentistry University of Fortaleza Fortaleza Brazil; ^2^ Department of Endodontics, Faculty of Dentistry University of Fortaleza Fortaleza Brazil; ^3^ Department of Materials and Ceramic Engineering, CICECO‐Aveiro Institute of Materials University of Aveiro Aveiro Portugal

## Abstract

**Background:**

Pulp calcification (PC) is a common imaging finding traditionally attributed to local factors or ageing. However, increasing evidence suggests that it may be associated with broader systemic biological disturbances. Clarifying this association may help determine whether dental pulp findings contribute to the interpretation of systemic health.

**Objectives:**

To evaluate whether imaging‐detected PC is associated with systemic conditions by synthesising evidence across diverse disorders and identifying recurring biological and methodological patterns.

**Methods:**

Comprehensive searches were conducted in EMBASE, ProQuest, PubMed, SciELO, Scopus, Web of Science, and the Virtual Health Library up to November 2025. Observational studies assessing associations between imaging‐detected PC and systemic conditions were included. Study quality and risk of bias were assessed using the Newcastle–Ottawa Scales and Joanna Briggs Institute tools. A mixed‐methods synthesis combined thematic analysis with random‐effects meta‐analysis. Associations were expressed as odds ratios (ORs) with 95% confidence intervals (CIs), and certainty of evidence was evaluated using GRADE.

**Results:**

Thirty‐three studies were included, comprising 19 057 patients. The primary meta‐analysis (*n* = 22) showed a statistically significant association between systemic conditions and PC (OR = 2.69; 95% CI: 2.04–3.54), although substantial heterogeneity was observed. Subgroup analyses identified a consistent association for diabetes mellitus, with no statistical heterogeneity (OR = 2.30; *I* ^2^ = 0.0%), while the atherosclerosis‐focused analysis showed the highest magnitude of association (OR = 4.70; *I* ^2^ = 0.0%). Broader cardiovascular disease conditions did not reach statistical significance (*p* = 0.155). Renal and genetic specialised conditions showed only exploratory evidence, based on two studies and rated as very low certainty. Thematic analysis suggested that PC may be associated with metabolic and vascular triggers, modulated by molar predilection and constrained by imaging resolution. The certainty of evidence ranged from low to very low across outcomes.

**Conclusion:**

Current evidence supports a statistically significant association between imaging‐detected PC and systemic metabolic and vascular conditions, particularly diabetes mellitus and atherosclerosis‐related outcomes. However, the evidence is predominantly observational and does not establish causality, temporality, prediction, or diagnostic utility. PC may represent an imaging finding associated with systemic dysregulation, but its biological significance remains uncertain and its interpretation should remain cautious and context‐dependent.

## 1 Introduction

Pulp calcification (PC) represents a significant endodontic challenge. This term serves as an umbrella definition for a spectrum of mineralising responses, ranging from discrete, nodular entities known as pulp stones to diffuse, generalised mineralisation termed pulp sclerosis or pulp canal obliteration (Goga et al. 2008). Understanding the constraints, variability, and unpredictability of its radiographic manifestations is fundamental for accurate diagnosis and clinical management (McCabe and Dummer 2012; Cruci et al. 2025). However, chromatic alterations, lack of response to sensibility tests, and complete radiographic pulp space obliteration do not constitute fully reliable indicators (McCabe and Dummer 2012). The variable composition of calcified deposits and their poorly understood mechanisms, potentially influenced by factors as diverse as patient diet (Bağcı et al. 2025), underscore the need for cautious interpretation.

Systemic conditions exert widespread effects throughout the body, including the oral cavity. This modulation of inflammatory, immunological, and metabolic behaviours is widely recognised in periodontology; conversely, its implications in endodontics are still being elucidated. Conditions such as cardiovascular diseases (CVD) and diabetes mellitus (DM) promote tissue damage through overlapping inflammatory pathways, immune dysfunction, or independent mechanisms (Susin et al. 2025). Similar dynamics are observed in autoimmune diseases, where oral manifestations often emerge among the first clinical signs (Saccucci et al. 2018). The interplay between genetic predisposition and environmental factors in these cases can culminate in chronic inflammation and significant progressive damage, both locally and systemically (Tavasoli et al. 2026).

Imaging remains a cornerstone of dental diagnosis. Although contemporary methods have enhanced the detection of discrete manifestations, significant limitations persist regarding the minimum detectable size and structural overlapping of PCs (Gonçalves et al. 2024). A meticulous image analysis is decisive for identifying subtle structural alterations and frequently represents the primary clinical evidence of pulp changes (McCabe and Dummer 2012). Additionally, the proper interpretation of these signs depends not only on perceiving the density and morphology of the deposits but also on understanding the biological factors modulating their formation (Cruci et al. 2025). Then, identifying a finding compatible with PC implies recognising that its radiographic expression is embedded in a biological context potentially influenced by systemic processes.

Gabardo et al. (2019) suggested a possible association between pulp stones and undiagnosed renal calculi, underscoring the clinical relevance of investigating such relationships. Similarly, Almadhoon et al. (2022) identified a significant association between pulp stones and CVDs, although no relationship with renal calculi was observed. Despite these efforts, the current evidence remains fragmented. It is therefore plausible to hypothesise that the pulp‐dentine complex and root canal systems may equally reflect these systemic dynamics. Under this biological rationale, the terminal microvascular architecture of the dental pulp suggests that the pulp‐dentine complex may reflect systemic disturbances through specific mineralising responses.

Thus, the present study evaluates whether imaging‐detected pulp calcification is associated with systemic conditions, while exploring whether pulpal mineralisation may be linked to broader patterns of host susceptibility. Evidence from a systematic review, thematic analysis, and meta‐analysis across diverse systemic conditions was pooled to identify recurring biological mechanisms and patterns.

## 2 Methods

### 2.1 Research Protocol and Reporting

The protocol was registered on the Prospective Register of Systematic Reviews (PROSPERO) platform (crd.york.ac.uk/PROSPERO/) and received the identification code: CRD420251241536. This manuscript was prepared in accordance with the Preferred Reporting Items for Systematic Reviews and Meta‐Analyses (PRISMA) (Page et al. 2021).

### 2.2 Research Question

The Population (P) – Exposure (E) – Comparator (C) – Outcome (O) (PECO) framework was used to formulate the guiding question: ‘What is the association between imaging‐detected pulp calcification (E) and systemic conditions (O) in dental patients (P) compared with those without calcification (C)?’ (Table 1).

**TABLE 1:** Structured PECO framework for research question construction.

Component	Definition	Specification
P	Population	Patients undergoing dental imaging examination, including panoramic radiograph, periapical radiograph, cone‐beam computed tomography (CBCT), or other imaging modalities
E	Exposure	Presence of pulp calcification and/or pulp stones confirmed by imaging
C	Comparator	Patients without pulp calcification or with different degrees/types of calcification
O	Outcome	Presence and strength of association between pulp calcifications and systemic diseases, including metabolic, cardiovascular, renal, autoimmune, inflammatory, genetic, endocrine, hepatic, respiratory, and others

### 2.3 Search Strategy

The base search strategy combined relevant Medical Subject Headings (MeSH) and key free‐text keywords identified during pilot testing, which were combined using the Boolean operators ‘AND’ and ‘OR’ (Table 2). The selection of terms was guided by the adopted ‘PECO’ framework (Table 1). The strategy was adapted for the advanced search tools of each selected database (Table S1).

**TABLE 2:** Thematic axes and search terms used in the systematic literature search, including Medical Subject Headings (MeSH) descriptors and keywords combined using Boolean operators.

Axis	Descriptors and keywords
Pulp Calcification	‘Dental Pulp Calcification’ OR ‘Dental Pulp Calcifications’ OR ‘Pulp Calcification’ OR ‘Pulp Calcifications’ OR ‘Dental Pulp Stone’ OR ‘Pulp Stone’ OR ‘Dental Pulp Stones’ OR ‘Pulp Stones’
Imaging	‘Diagnostic Imaging’ OR ‘Radiography’ OR ‘Radiography, Dental’ OR ‘Imaging, Diagnostic’ OR ‘Medical Imaging’ OR ‘Imaging, Medical’ OR ‘Diagnostic X‐Ray’ OR ‘X‐Ray’ OR ‘X Ray’ OR ‘Diagnostic X Ray’ OR ‘X‐Rays, Diagnostic’ OR ‘Dental Radiograph’ OR ‘Dental Radiography’ OR ‘CBCT’ OR ‘Cone Beam Computed Tomography’ OR ‘Panoramic Radiograph’
Conditions	‘Systemic Disease’ OR ‘Syndrome’ OR ‘Genetic Disorder’ OR ‘Endocrine Disorder’ OR ‘Metabolic Disorder’ OR ‘Autoimmune Disease’ OR ‘Inflammatory Disease’ OR ‘Cardiovascular Disease’ OR ‘Hypertension’ OR ‘Hyperlipidemia’ OR ‘Atherosclerosis’ OR ‘Diabetes Mellitus’ OR ‘Obesity’ OR ‘Renal Disease’ OR ‘Chronic Kidney Disease’ OR ‘Hepatic Disease’ OR ‘Respiratory Disease’
Base Strategy	((‘Dental Pulp Calcification’ OR ‘Dental Pulp Calcifications’ OR ‘Pulp Calcification’ OR ‘Pulp Calcifications’ OR ‘Dental Pulp Stone’ OR ‘Pulp Stone’ OR ‘Dental Pulp Stones’ OR ‘Pulp Stones’) AND (‘Diagnostic Imaging’ OR ‘Radiography’ OR ‘Radiography, Dental’ OR ‘Imaging, Diagnostic’ OR ‘Medical Imaging’ OR ‘Imaging, Medical’ OR ‘Diagnostic X‐Ray’ OR ‘X‐Ray’ OR ‘X Ray’ OR ‘Dental Radiograph’ OR ‘Dental Radiography’ OR ‘CBCT’ OR ‘Cone Beam Computed Tomography’ OR ‘Panoramic Radiograph’)) AND (‘Systemic Disease’ OR ‘Syndrome’ OR ‘Genetic Disorder’ OR ‘Endocrine Disorder’ OR ‘Metabolic Disorder’ OR ‘Autoimmune Disease’ OR ‘Inflammatory Disease’ OR ‘Cardiovascular Disease’ OR ‘Hypertension’ OR ‘Hyperlipidemia’ OR ‘Atherosclerosis’ OR ‘Diabetes Mellitus’ OR ‘Obesity’ OR ‘Renal Disease’ OR ‘Chronic Kidney Disease’ OR ‘Hepatic Disease’ OR ‘Respiratory Disease’)

### 2.4 Eligibility Criteria

Included studies comprised observational research, such as cross‐sectional, case–control, cohort, and other original investigations reporting associations between imaging‐detected PC and systemic diseases. Both randomised and non‐randomised study types were considered to ensure a comprehensive evidence mapping. Studies were excluded if they lacked clear imaging‐based evidence of PC, such as clinical assessments without radiographic confirmation; focused solely on endodontic treatment outcomes without reporting systemic data; or were in vitro, in silico, in vivo (animal), or ex vivo (cadaveric) studies. Reviews, commentaries, editorials, conference abstracts, and duplicate publications were also excluded.

### 2.5 Data Sources

Comprehensive searches were independently performed by two calibrated reviewers experienced in endodontics and diagnostic imaging across Excerpta Medica dataBASE (EMBASE), Medline/PubMed, ProQuest (Dissertations & Theses), Scientific Electronic Library Online (SciELO), Scopus, Web of Science (WoS), and Virtual Health Library (VHL). Grey literature was partially assessed during the ProQuest search. Additionally, reference lists of included studies were screened to identify relevant reports not captured by the electronic searches. Disagreements were resolved through research team consensus.

### 2.6 Study Selection and Data Extraction

A two‐phase selection process was conducted through Rayyan (rayyan.ai/), independently and in a blinded manner, after prior consensus team calibration. Titles and abstracts were screened first, followed by full‐text review to confirm eligibility. Data were extracted using standardised, pilot‐tested spreadsheets capturing publication details, clinical features, imaging findings, exposures/outcomes, and overall study design characteristics.

### 2.7 Study Quality and Risk of Bias Assessment

Methodological quality was appraised in pairs, independently and in a blinded manner, after consensus calibration. Case–control and cohort studies were evaluated using the Newcastle‐Ottawa Scale (NOS) (Wells et al. 2013), assessing selection, comparability, and outcome/exposure. Cross‐sectional studies were assessed with the Joanna Briggs Institute (JBI) Critical Appraisal Checklist (Moola et al. 2017), covering inclusion criteria, confounding factors, and statistical analysis. Studies scoring 7–9 stars on the NOS (with at least one star in the comparability domain) or 7–8 affirmative responses on the JBI checklist were classified as high methodological quality. Those meeting 4–6 criteria or failing to adequately address comparability were classified as moderate methodological quality, whereas studies fulfilling ≤ 3 criteria were considered to have low methodological quality. Disagreements were resolved through discussion with the full review team. To comply with systematic review standards requiring explicit consideration of risk of bias, the methodological quality assessments were subsequently operationalised into domain‐level risk‐of‐bias judgements. Limitations identified within the NOS and JBI domains were mapped to corresponding sources of bias, including selection, confounding, and measurement bias, and were incorporated into the interpretation of individual study findings. This approach enabled a transparent appraisal of risk of bias while preserving the design‐specific characteristics of each assessment tool.

### 2.8 Outcomes Measures

The primary outcome was defined as the presence or absence of systemic conditions (O) in individuals with imaging‐detected PC (E). Effect estimates including odds ratios (ORs), relative risks (RRs), or prevalence ratios with 95% confidence intervals (CIs) were extracted to quantify this association. Secondary outcomes included variables potentially modulating the primary association, such as demographic and clinical correlates (e.g., age, sex, and comorbidities), imaging‐based descriptors (e.g., number, size, and distribution), and technical parameters influencing detection accuracy, including imaging modality and acquisition settings.

### 2.9 Data Synthesis

Evidence was synthesised using a mixed‐methods framework, integrating qualitative thematic analysis and quantitative meta‐analysis.

#### 2.9.1 Thematic Analysis

Narrative synthesis was complemented by thematic analysis for studies reporting continuous outcomes or incompatible effect measures, following Braun and Clarke's (2006) framework: (i) familiarisation; (ii) initial code generation; (iii) searching for themes; (iv) reviewing themes; (v) defining and naming themes; and (vi) producing the report. This approach helped identify overarching conceptual patterns when statistical pooling was unfeasible, ensuring a more robust interpretation of the entire evidence base.

#### 2.9.2 Meta‐Analysis

Meta‐analyses were conducted in RStudio (version 2023.12.1; Posit Software, PBC, Boston, USA) using random‐effects models for dichotomous outcomes, implemented with the ‘metafor’ (Viechtbauer 2010) and ‘meta’ (Schwarzer et al. 2015) packages. The primary pooled analysis was intentionally designed as a broad association model to determine whether imaging‐detected PCs were more frequent among individuals with systemic conditions as a general exposure category. This decision was based on the shared conceptual premise that several systemic conditions may influence mineralising responses through inflammatory, vascular, metabolic, or immunological pathways. Nevertheless, because these conditions differ substantially in pathophysiology, the pooled estimate was not interpreted as representing a single disease‐specific mechanism. For this reason, subgroup analyses were prioritised to identify more coherent biological signals and to avoid overgeneralisation of the global effect estimate. The Hartung‐Knapp (HK) adjustment was applied to calculate odds ratios (ORs) and 95% confidence intervals (CIs) for the pooled effect estimates to account for between‐study heterogeneity and provide a more conservative statistical inference. Patient‐level analyses were adopted as the primary unit of analysis to mitigate clustering effects inherent in tooth‐level data. Multiple teeth from the same individual are biologically and statistically non‐independent; therefore, tooth‐level pooling could artificially inflate sample size, narrow confidence intervals, and overestimate the precision of the association. Patient‐level data were thus prioritised to ensure that ORs and CIs reflected systemic biological associations at the individual level. Tooth‐level information was retained narratively whenever available, particularly to describe anatomical distribution patterns such as molar predilection. Statistical heterogeneity was assessed using the I^2^ statistic and Cochran's Q test, with a significance threshold of *p* < 0.10. Sensitivity analyses employed a leave‐one‐out approach to evaluate the robustness of the pooled estimates and identify studies with disproportionate influence on the results. Publication bias was evaluated through visual inspection of funnel plots and supported by formal statistical testing using Begg's rank correlation (Begg and Mazumdar 1994) and Egger's linear regression (Egger et al. 1997) tests whenever at least ten studies were available, in accordance with established recommendations.

### 2.10 Certainty Assessment

The certainty of the evidence was assessed using the Grading of Recommendations Assessment, Development and Evaluation (GRADE) approach (Schünemann et al. 2013) across five domains: risk of bias, inconsistency, indirectness, imprecision, and publication bias. Evidence was downgraded when serious or very serious concerns were identified within any domain. Inconsistency was evaluated based on the degree of statistical heterogeneity and variability of effect estimates. Indirectness was assessed according to the alignment between study populations, exposures, and outcomes with the predefined PECO framework. Imprecision was considered using confidence interval width and sample size. Publication bias was assessed visually via funnel plots and, when feasible, through formal statistical tests. Upgrading the evidence was considered in cases of large magnitude of effect.

## 3 Results

A total of 291 records were retrieved through comprehensive database searches. After removing 121 duplicates, 170 records remained for title and abstract screening. Forty‐four reports were sought for retrieval; however, one full text was unavailable. Consequently, 43 reports were assessed for eligibility, of which 11 were excluded based on specific criteria (Table S2). Additionally, 8 records were identified through citation searching (Table S3); of these, 7 were excluded and 1 was included. Ultimately, 33 studies were definitively included in the review. The screening process is illustrated in Figure 1.

**FIGURE 1:**
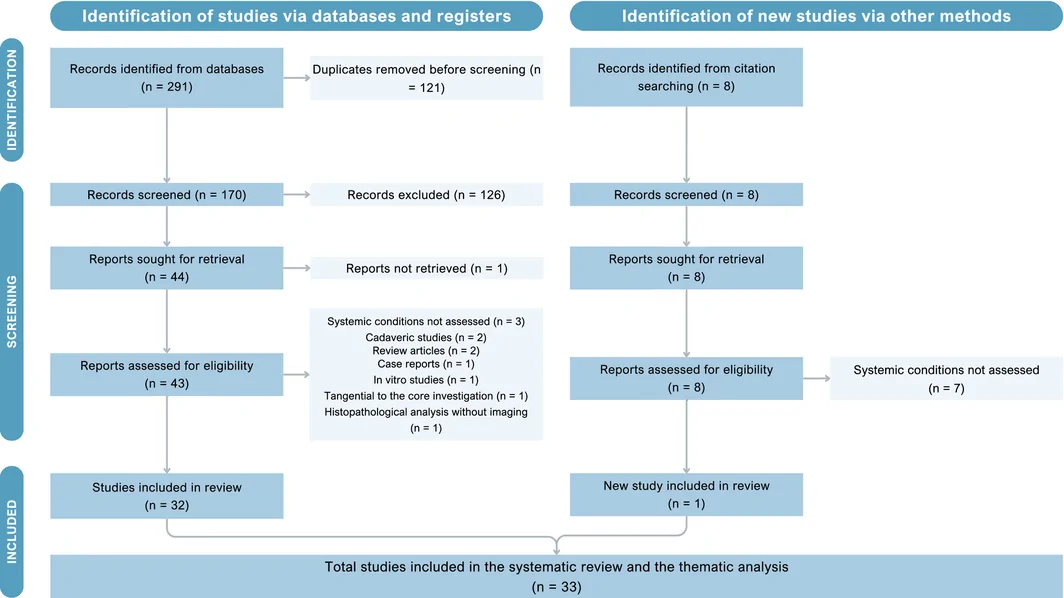
PRISMA flow diagram of the study selection process for the systematic review on pulp calcification and systemic diseases.

### 3.1 Overview of Included Studies

The included investigations reported data on the presence of PCs, pulp space narrowing, and/or pulp obliteration, showing heterogeneous results regarding their association with systemic conditions. A significant subset of studies evaluated CVDs, DM, and renal diseases, while others addressed genetic syndromes, other metabolic disorders, and chronic medication use. Most investigations utilised panoramic and periapical radiographs, with a limited number of studies employing cone‐beam computed tomography (CBCT) or histopathological analysis. Several reports indicated a higher involvement of molars, particularly first molars, in addition to an increased prevalence of calcifications in advanced age groups (Table 3). These studies exhibited a broad geographical distribution across 10 countries: Türkiye (*n* = 9), India (*n* = 7), Saudi Arabia (*n* = 6), Iran (*n* = 3), Brazil (*n* = 2), United States of America (USA) (*n* = 2), Chile (*n* = 1), Germany (*n* = 1), Sweden (*n* = 1), and Thailand (*n* = 1) (Figure 2).

**TABLE 3:** Demographic characteristics and methodology features of the included studies reporting pulp calcifications in relation to systemic conditions.

Study	Systemic condition(s)	Imaging modality	Outcome assessed	Analysis unit	Comparison groups	Sample size
Näsström et al. (1985), Sweden	Renal diseases (Transplant, HD)	Periapical and Bitewing	Pulp narrowing; Pulp obliteration	Patient	Transplant vs. Immunosuppressed vs. HD	51
Moura and Paiva (1987), Brazil	Coronary atherosclerosis	Periapical	Free pulpal calcifications	Patient and Tooth	Atherosclerosis vs. Ischemic controls	50 patients; 570 teeth
Edds et al. (2005), USA	CVD	Periapical	Pulp stones	Patient	CVD vs. No CVD	55
Bauss et al. (2008), Germany	Marfan syndrome	Bitewing	Pulp stones; Pulp obliteration	Patient and Tooth	Marfan vs. Controls	121
Gulsahi et al. (2009), Türkiye	Multiple systemic diseases	Periapical	Pulp stones	Patient and Tooth	Systemic vs. Healthy	519 patients; 13 474 teeth
Kansu et al. (2009), Türkiye	ESRD (Haemodialysis, Transplant)	Panoramic and Periapical	Pulp narrowing; Pulp stones	Patient	Haemodialysis vs. Transplant	60
Şener et al. (2009), Türkiye	CVD	Periapical and Bitewing	Pulp chamber calcifications	Patient and Tooth	CVD vs. Non‐CVD	536 patients; 15 326 teeth
Horsley et al. (2009), USA	CAC	Panoramic	Pulpal calcifications	Patient	CAC (+) vs. CAC (−)	247 radiographs
Nayak et al. (2010), India	CVS, DM, Autoimmune	Periapical and RVG	Pulp stones	Patient and Tooth	Systemic groups vs. Control	150 patients; 4296 teeth
Ezoddini‐Ardakani et al. (2011), Iran	CAS	Panoramic	Pulp stones	Patient and Tooth	CAS vs. No CAS	61
Kanjanabuch et al. (2011), Thailand	CAPD	Panoramic and Bitewing	Pulp calcification	Patient (mean % of teeth)	CAPD vs. Controls	30 (13 CAPD, 17 Control)
Vidyullatha et al. (2011), India	ESRD	Panoramic and Periapical	Pulp calcification	Patient	ESRD (Descriptive)	40
Khojastepour et al. (2013), Iran	CVD	Panoramic	Dental pulp calcification	Patient	CVD vs. Healthy	122
Patil and Sinha (2013), India	ESRD	Panoramic and Periapical	Pulp narrowing; Stones	Patient	Association with CAC	112
Bains et al. (2014), India	CVD, Lithiasis	Bitewing	Pulp stones	Patient and Tooth	CVD/Lithiasis vs. Control	500 patients; 5333 teeth
Özakar İlday et al. (2014), Türkiye	Multiple systemic diseases	Panoramic	Pulp stones	Patient and Tooth	Multiple factors	4798
Talla et al. (2014), India	CVS, DM, Gastritis	Periapical	Pulp stones	Patient and Tooth	Systemic vs. Control	2000
Çağlayan et al. (2015), Türkiye	CRF (Renal)	CBCT	Pulp calcification	Patient	CRF vs. Control	30
Swathy et al. (2015), India	Cardiac disease	Periapical and Panoramic	Pulp stones	Patient	Cardiac vs. Control	60
Alsweed et al. (2019), Saudi Arabia	CAC	Panoramic	CAC & Pulp stones	Patient	Pulp stones vs. CAC	2013
Mathew et al. (2019), Saudi Arabia	CVD, DM	Panoramic, Bitewing, and Periapical	Pulp stones	Patient	Compromised vs. Fit	1030
Simsek et al. (2019), Türkiye	Obesity	Panoramic	Pulp stone	Patient	BMI groups (Obese vs. Normal)	186
Gunen Yilmaz et al. 2019, Türkiye	CAC	Panoramic	Pulp stone	Patient and Tooth	CAC (+) vs. CAC (−)	60
Araya et al. (2020), Chile	DM	Periapical	Chamber calcifications	Tooth	DM vs. Non‐DM	617
Srivastava et al. (2020), Saudi Arabia	CVD, Diabetes Mellitus	CBCT	Pulp stones	Patient and Tooth	CVD/DM vs. Control	229
Jawahar et al. (2021), India	Hypertension, Hyperlipidaemia	Periapical	Pulp stones	Patient	Disease correlation	70
Ozsin Ozler et al. 2022, Türkiye	IBMFS	Panoramic	Pulp stones	Patient	Descriptive	27
Alamoudi et al. (2023), Saudi Arabia	Multiple systemic diseases	Periapical and Bitewing	Pulp canal calcification	Patient	Cases vs. Controls	200
de Souza Romano et al. 2024, Brazil	Multiple kidney stones	Panoramic and Periapical	Pulp stones	Patient	Pulp stones vs. No Pulp stones	490
Kaabi et al. (2024), Saudi Arabia	Systemic + Medications	CBCT	Pulp stones	Patient and Tooth	Systemic vs. None	644
Beytaş Alğan et al. (2025), Türkiye	Systemic diseases	Panoramic	Pulp stones	Patient	Diseased vs. Healthy	4050
Baghestani et al. (2025), Iran	DM	Panoramic	Pulp stones; CAC	Patient	DM vs. Healthy	407
Fairozekhan et al. (2025), Saudi Arabia	Multiple systemic diseases	Panoramic	Pulp stones	Patient and Tooth	Medical history groups	153

Abbreviations: BMI, Body Mass Index; CAC, Carotid Artery Calcification; CAPD, Continuous Ambulatory Peritoneal Dialysis; CAS, Coronary Artery Stenosis; CBCT, Cone‐Beam Computed Tomography; CRF, Chronic Renal Failure; CVD, Cardiovascular Disease; CVS, Cardiovascular System; DM, Diabetes Mellitus; ESRD, End‐Stage Renal Disease; HD, Haemodialysis; IBMFS, Inherited Bone Marrow Failure Syndromes; NR, Not Reported; RVG, Radiovisiography.

**FIGURE 2:**
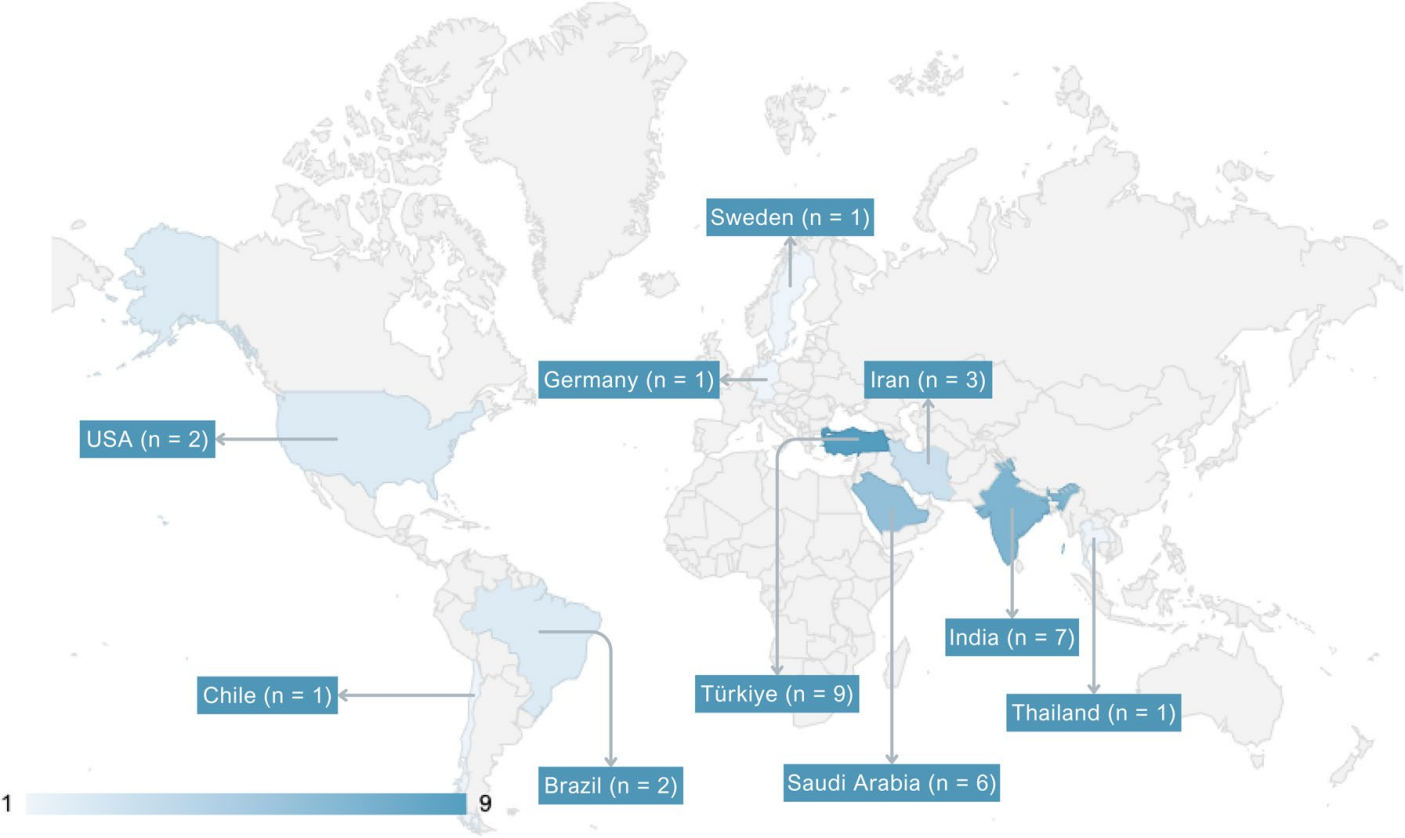
Data chart summarising the geographical distribution of the 33 included studies.

Sample sizes ranged from small clinical cohorts to large imaging series, involving a cumulative total of 19 057 patients and 175 241 teeth. Reporting patterns regarding the unit of analysis were notably inconsistent across the evidence base. While quantifiable patient‐level data were available for 30 studies (*n* = 18 951), several investigations failed to provide raw tooth‐level counts or presented internal numerical discrepancies. Furthermore, the use of continuous mean values and percentages in some reports, coupled with the omission of patient‐to‐tooth ratios in others, prevented a full tooth‐level aggregate. Consequently, patient‐level data were prioritised for the primary meta‐analysis to ensure statistical reliability and mitigate the effects of reporting heterogeneity.

### 3.2 Study Quality and Risk of Bias Assessment

The methodological quality was appraised using design‐specific instruments (Tables S4–S6). Only five studies were classified as having a low risk of bias (Kanjanabuch et al. 2011; Khojastepour et al. 2013; Simsek et al. 2019; Alamoudi et al. 2023; Baghestani et al. 2025), which corresponds to high methodological quality as they presented clearly defined inclusion criteria, adequate measurement of exposures and outcomes, and appropriate strategies for controlling confounding factors. The majority of the studies (*n* = 24) were categorised as moderate risk (Näsström et al. 1985; Moura and Paiva 1987; Edds et al. 2005; Bauss et al. 2008; Gulsahi et al. 2009; Şener et al. 2009; Horsley et al. 2009; Nayak et al. 2010; Ezoddini‐Ardakani et al. 2011; Vidyullatha et al. 2011; Bains et al. 2014; Özakar İlday et al. 2014; Talla et al. 2014; Çağlayan et al. 2015; Swathy et al. 2015; Gunen Yilmaz et al. 2019; Araya et al. 2020; Srivastava et al. 2020; Jawahar et al. 2021; Ozsin Ozler et al. 2022; de Souza Romano et al. 2024; Kaabi et al. 2024; Beytaş Alğan et al. 2025; Fairozekhan et al. 2025). These works demonstrated adequate sample definitions and outcome measurements but frequently exhibited limitations in identifying and controlling confounders, as well as a lack of blinding during outcome assessment. Four studies were classified as high risk of bias (Kansu et al. 2009; Patil and Sinha 2013; Alsweed et al. 2019; Mathew et al. 2019), which corresponds to low methodological quality, primarily due to non‐representative samples, inadequate confounding control, incomplete follow‐up, or lack of blinding (Figure 3).

**FIGURE 3:**
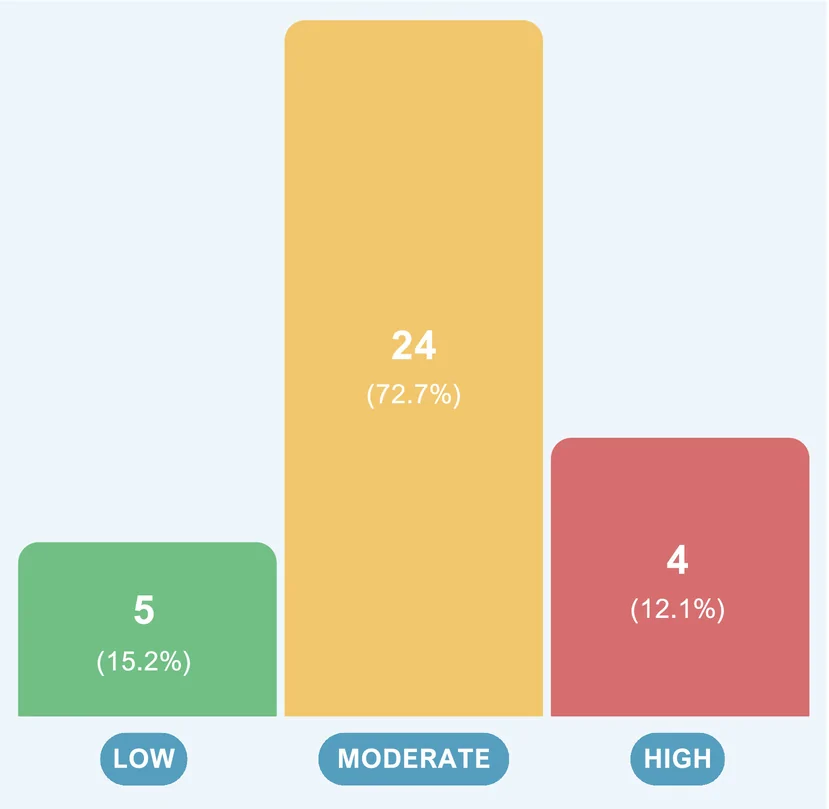
Distribution of the overall risk of bias across the 33 included studies.

To facilitate a comprehensive synthesis of the study quality assessment and the translation into risk of bias, the methodological findings were summarised into four harmonised thematic domains: (1) Selection (sample definition), (2) Confounding (comparability), (3) Measurement (blinding/outcome), and (4) Statistical analysis, illustrated in Figure 4. This allowed the identification of systematic vulnerabilities across the 33 included studies whilst preserving the original criteria of each tool. Overall, statistical analysis was the most robust domain, with 28 (84.8%) studies classified as low risk. Selection (sample definition) demonstrated low risk in 22 (66.7%) studies. Conversely, the most significant methodological vulnerability was identified in the confounding (comparability) domain, where 20 (60.6%) studies presented a moderate or high risk of bias due to insufficient control of extraneous variables. Regarding measurement (blinding/outcome), 23 (69.7%) studies were assessed as moderate or high risk, primarily reflecting the lack of blinded radiographic assessment and standardised outcome reporting. In summary, the evidence base presented a predominantly moderate methodological quality. The observed heterogeneity in selection processes, exposure and outcome measurement, and the identification of confounding factors constituted the primary methodological limitations. In particular, smoking, chronic medication use, dietary patterns, and genetic predisposition may influence mineral metabolism, vascular inflammation, or ectopic calcification tendencies. Unequal distribution of these variables may inflate the observed OR by attributing calcification to systemic disease rather than shared background risk factors, whereas controls with undiagnosed or related conditions may attenuate the association. Domain‐based analysis revealed recurring patterns: in studies evaluated via the NOS, selection domains were generally better documented than comparability and outcome domains. For studies appraised via the JBI checklist, inclusion criteria and setting characterisation were usually adequate; however, explicit identification and management of confounding factors were frequently absent or unclear, contributing to the moderate risk rating.

**FIGURE 4:**
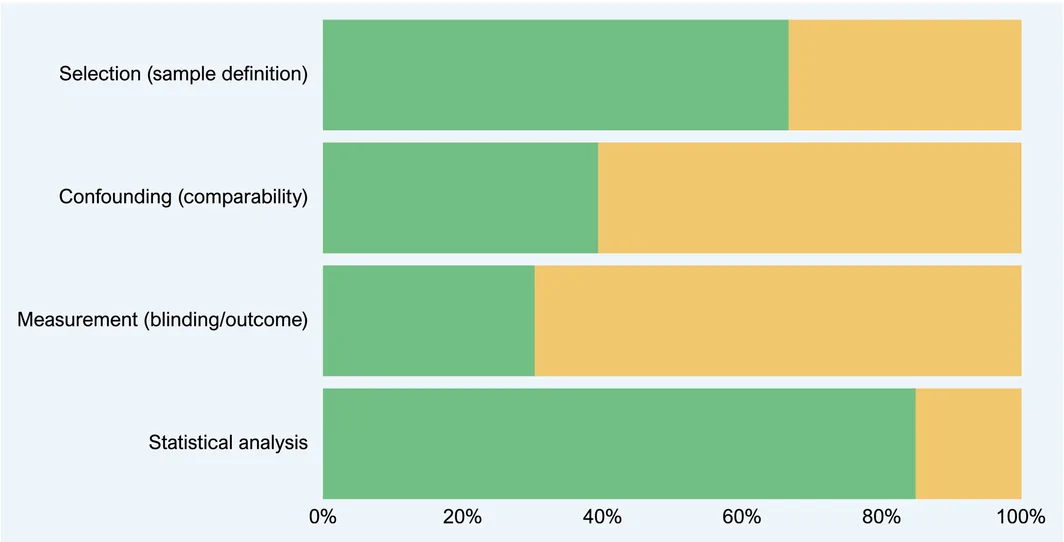
Risk of bias summary based on harmonised methodological domains. Each study was evaluated across four main thematic domains: Selection (sample definition), confounding (comparability), measurement (blinding and outcome assessment), and statistical analysis. Colour coding indicates low (green) and moderate (yellow) risk of bias for each domain.

### 3.3 Thematic Analysis

The detailed coding process and thematic refinement are structured in Table S7. The analysis revealed recurring patterns organised into three primary axes, visually summarised in Figure 5. The first axis, *Biological Triggers*, integrates evidence associating PCs and pulp space narrowing with metabolic, vascular, and pharmacological systemic conditions. This theme suggests that the dental pulp may function as a sensitive microenvironment in which systemic dysregulation is associated with local mineralising responses. The second axis, *Local Modulators*, refers to anatomical vulnerability, reflected in the consistent predilection for molars. This pattern is underpinned by the synergy between pulp volume and vascularity, which may condition the phenotypic expression of systemic background factors. The third axis, *Observational Constraints*, addresses the impact of technical limitations on the detection of the association. It underscores how imaging resolution thresholds and heterogeneous definitions drive the variability in prevalence estimates, conditioning the observability of the phenomenon.

**FIGURE 5:**
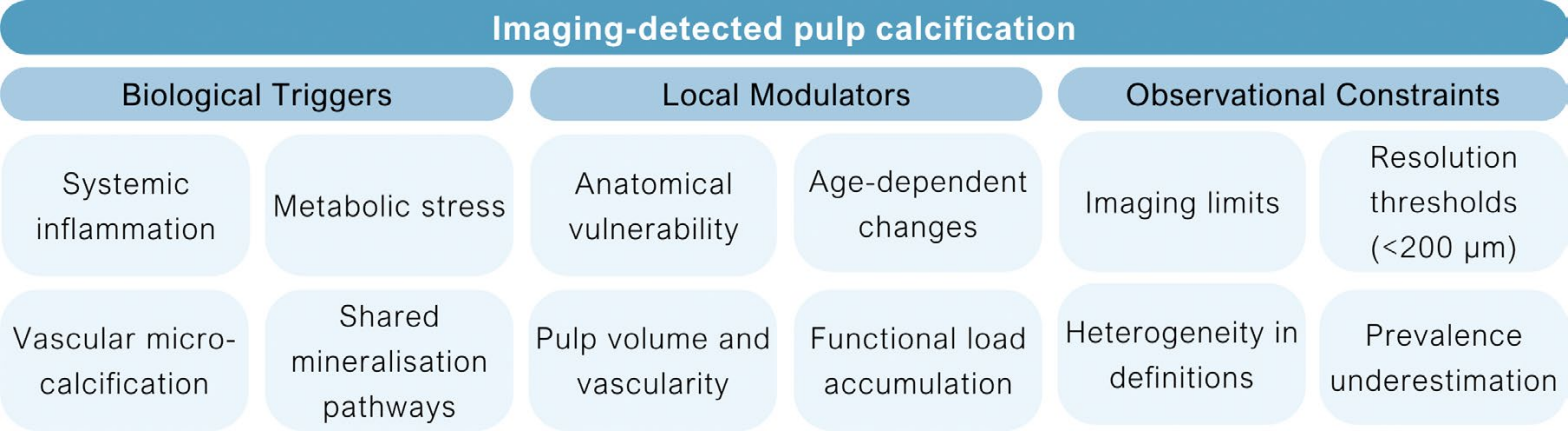
Determinants of the association between imaging‐detected pulp calcification and systemic conditions.

### 3.4 Meta‐Analyses

Meta‐analysis was conducted only when minimal clinical homogeneity, compatible dichotomous data, and conceptual comparability of outcomes were present. Given the wide heterogeneity of systemic conditions, diagnostic methods, and units of analysis, comparisons were stratified into subgroups to enhance clinical interpretability. Where applicable, leave‐one‐out sensitivity analyses reinforced the robustness of the main and larger subgroup estimates, demonstrating that these findings were not driven by single studies (Figures 6, 7).

**FIGURE 6:**
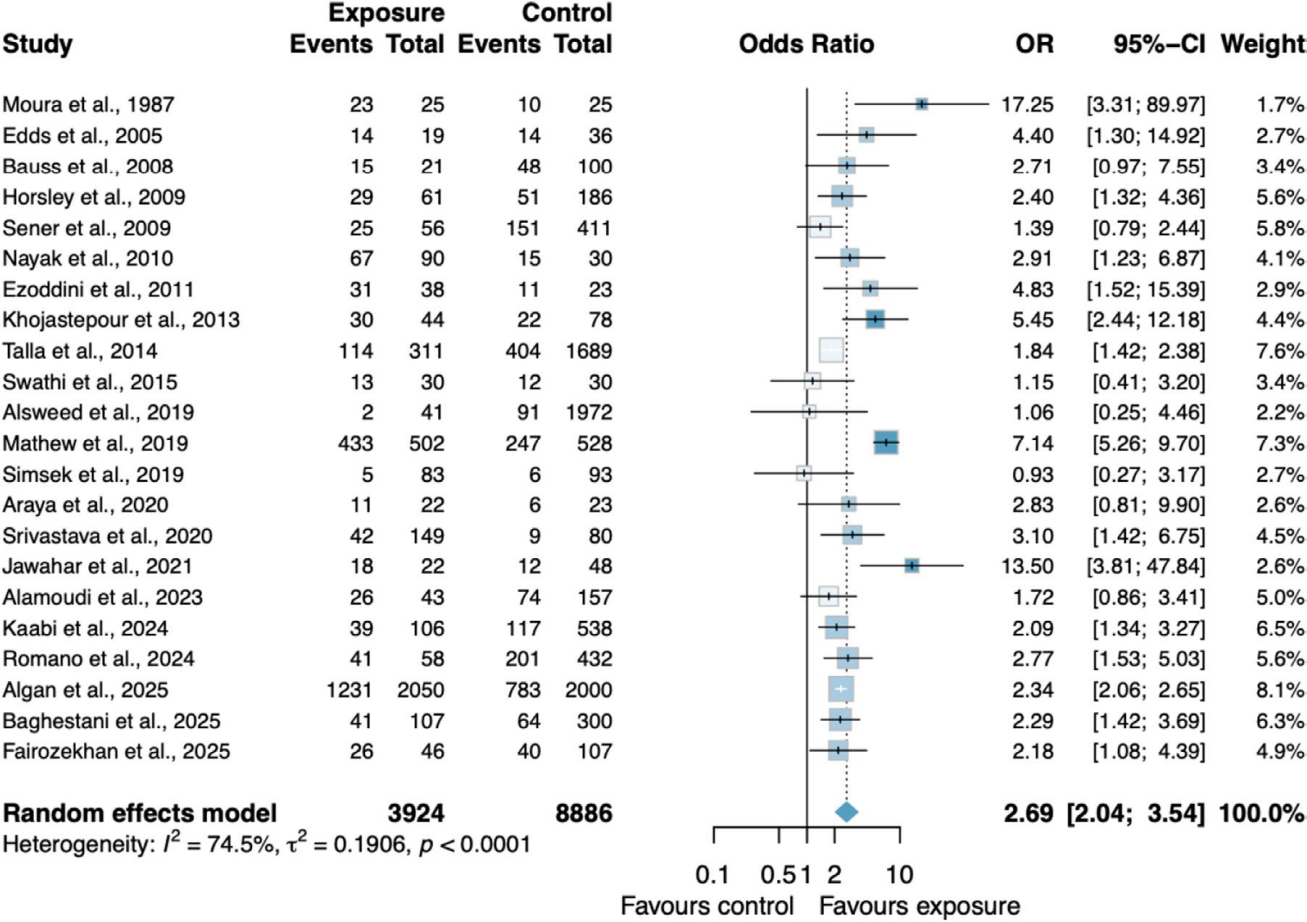
Forest plot of the association between systemic conditions and pulp calcification (*n* = 22 studies).

**FIGURE 7:**
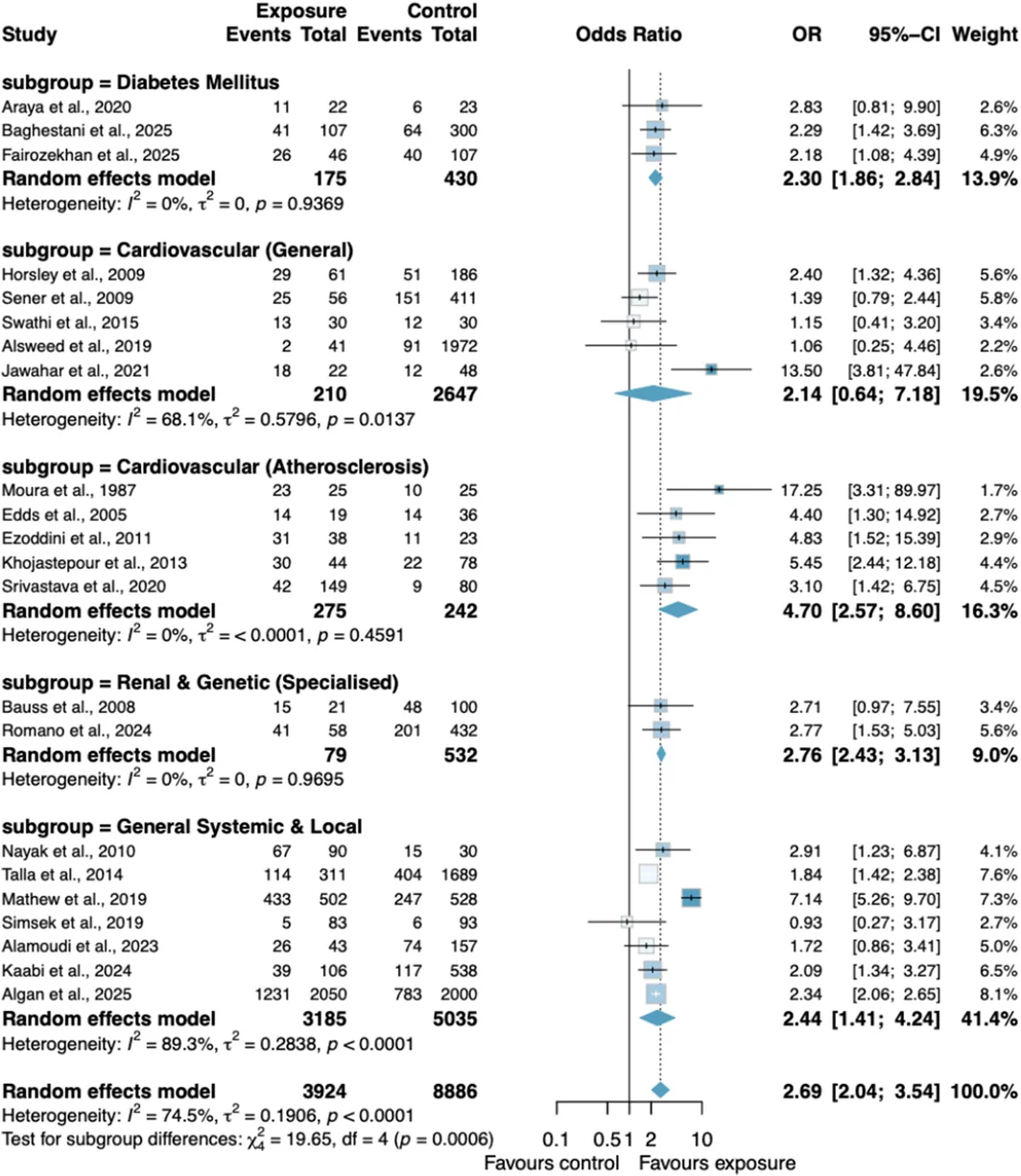
Subgroup meta‐analysis of pulp calcification occurrence across different systemic categories.

#### 3.4.1 General Systemic Conditions

Twenty‐two studies were eligible for the primary meta‐analysis, comparing individuals with various systemic conditions to healthy controls regarding the presence of PCs (Moura and Paiva 1987; Edds et al. 2005; Bauss et al. 2008; Horsley et al. 2009; Şener et al. 2009; Nayak et al. 2010; Ezoddini‐Ardakani et al. 2011; Khojastepour et al. 2013; Talla et al. 2014; Swathy et al. 2015; Alsweed et al. 2019; Mathew et al. 2019; Simsek et al. 2019; Araya et al. 2020; Srivastava et al. 2020; Jawahar et al. 2021; Alamoudi et al. 2023; Kaabi et al. 2024; de Souza Romano et al. 2024; Beytaş Alğan et al. 2025; Baghestani et al. 2025; Fairozekhan et al. 2025). The random‐effects model indicated a statistically significant association between systemic diseases and pulp stones, with an OR of 2.69 (95% CI: 2.04 to 3.53; *p* < 0.0001). Substantial heterogeneity was observed (*I* ^2^ = 74.5%), which was considered expected given the clinical diversity of exposures and general variations among the studies. Disease‐specific subgroup analyses were considered more clinically informative for interpreting the biological plausibility of the findings. Nevertheless, the direction of effect was consistent, with most studies favouring a higher occurrence of PCs in the exposed group (Figure 7). The leave‐one‐out sensitivity analysis demonstrated substantial robustness of the global estimate. Sequential exclusion of individual studies did not alter the statistical significance of the pooled effect, nor did it meaningfully change its magnitude. OR estimates varied within a narrow range (2.57 to 2.79) and remained consistently above unity, indicating that the findings were not driven by any single influential study (Figure S1).

#### 3.4.2 Diabetes Mellitus

Three studies specifically evaluated the association between DM and PCs (Araya et al. 2020; Baghestani et al. 2025; Fairozekhan et al. 2025). The random‐effects model revealed a consistent and statistically significant association, with an OR of 2.30 (95% CI: 1.86 to 2.84; *p* < 0.0001). No heterogeneity was detected (*I* ^2^ = 0.0%), suggesting high consistency among the available evidence, although this estimate should be interpreted considering the limited number of studies in this subgroup (Figure 7). Leave‐one‐out sensitivity analysis confirmed the stability of this association, with no individual study exerting a meaningful influence on the pooled estimate (Figure S2).

#### 3.4.3 Cardiovascular Conditions

Studies evaluating CVD were stratified into two subgroups to isolate specific patterns (Figure 7). Ten studies contributed to the CVD analyses. (i) *General cardiovascular subgroup*: Five studies focused on general CVD conditions (Horsley et al. 2009; Şener et al. 2009; Swathy et al. 2015; Alsweed et al. 2019; Jawahar et al. 2021). The pooled analysis yielded an OR of 2.12 (95% CI: 0.65 to 6.87; *p* = 0.155), with moderate heterogeneity (*I* ^2^ = 68.1%). This subgroup did not reach statistical significance, indicating that broad or non‐specific cardiovascular diagnoses do not provide a reliable association signal for PCs. (ii) *Atherosclerosis‐focused subgroup*: Five studies evaluated the association between atherosclerotic markers or vascular calcification and pulp stones (Moura and Paiva 1987; Edds et al. 2005; Ezoddini‐Ardakani et al. 2011; Khojastepour et al. 2013; Srivastava et al. 2020). The random‐effects model demonstrated a strong and statistically significant association, with an OR of 4.70 (95% CI: 2.57 to 8.60; *p* < 0.0001). No heterogeneity was observed (*I* ^2^ = 0.0%), suggesting a consistent relationship between vascular calcification and PCs. Leave‐one‐out sensitivity analysis showed that the pooled estimate remained stable and statistically significant throughout all sequential exclusions (Figures S3, S4).

#### 3.4.4 Renal and Genetic Specialised Conditions

The quantitative synthesis for renal and genetic/specialised conditions included only two studies (Bauss et al. 2008; de Souza Romano et al. 2024) and was intended only to explore potential signals. Although the random‐effects model yielded a statistically significant estimate (OR = 2.76; 95% CI: 2.43–3.13; *p* = 0.006) (Figure 7), this subgroup combined distinct clinical entities and should not be interpreted as clinically homogeneous. Therefore, these findings should be regarded as hypothesis‐generating and not as evidence of a shared mechanism or generalisable association with broader systemic disease.

#### 3.4.5 Non‐Specific and Multiple Systemic Conditions

A specific subset of seven studies evaluated populations characterised by broad or multiple systemic conditions that did not fit into mutually exclusive disease categories (Nayak et al. 2010; Talla et al. 2014; Mathew et al. 2019; Simsek et al. 2019; Alamoudi et al. 2023; Kaabi et al. 2024; Beytaş Alğan et al. 2025). This subgroup yielded a statistically significant pooled OR of 2.44 (95% CI: 1.41–4.24; *p* < 0.0001) (Figure 7). Nonetheless, substantial heterogeneity was detected (*I* ^2^ = 89.3%). The leave‐one‐out sensitivity analysis revealed that Mathew et al. (2019) acted as a significant source of statistical inconsistency (Figure S5). Its exclusion reduced the heterogeneity parameters significantly and adjusted the pooled estimate to 2.11 (95% CI: 1.75–2.56), suggesting that large retrospective datasets with non‐specific inclusion criteria may introduce variance into global estimates.

### 3.5 Publication Bias

Publication bias was evaluated through visual inspection of funnel plots and formal statistical tests. For the primary meta‐analysis (*n* = 22), the funnel plot showed an approximately symmetrical distribution of studies around the pooled effect estimate (Figure 8). This visual assessment was supported by formal statistical testing, as neither Egger's linear regression test (*p* = 0.704) nor Begg and Mazumdar's rank correlation test (*p* = 0.176) indicated statistically significant funnel plot asymmetry. These findings combined suggest a low likelihood that the observed associations are influenced by publication bias. Formal asymmetry tests were not conducted for the subgroup analyses due to the limited number of studies per subgroup (*k* < 10). The evaluation of these analyses relied primarily on the robustness demonstrated in the leave‐one‐out sensitivity analyses.

**FIGURE 8:**
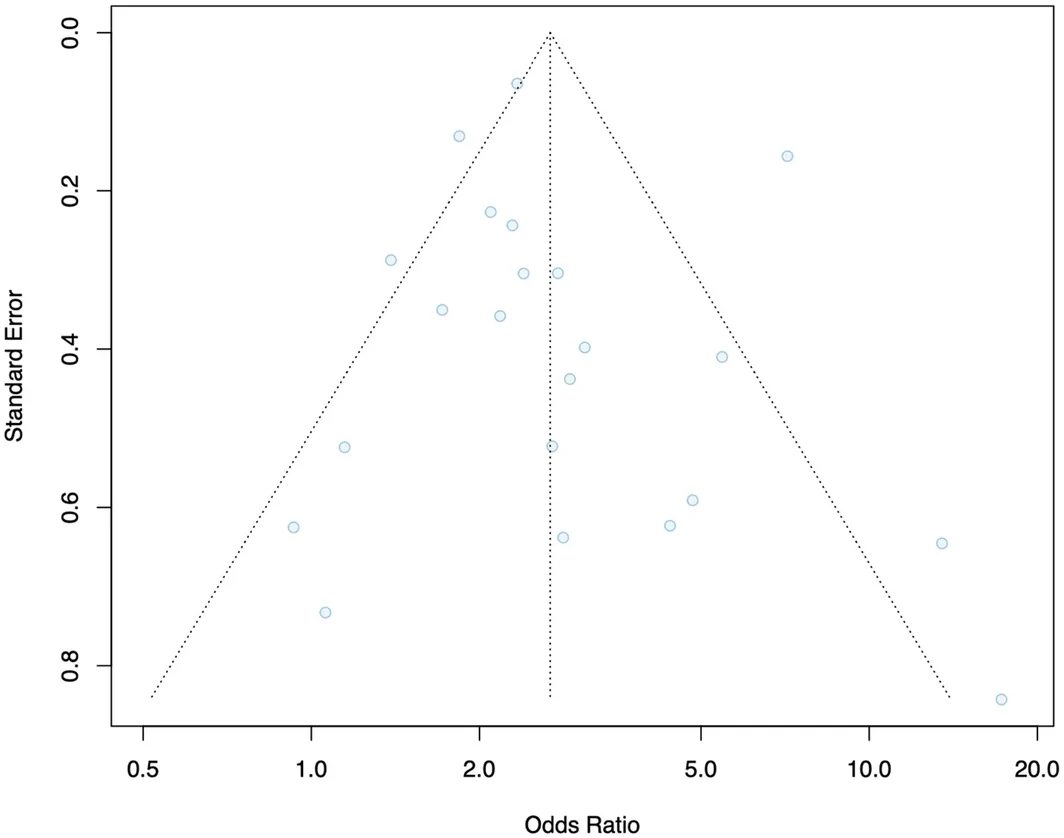
Funnel plot showing the distribution of the 22 studies around the pooled effect estimate.

### 3.6 Certainty of Evidence

The certainty of evidence was assessed using the GRADE approach. In response to the observational nature of the evidence and the methodological limitations identified across studies, the certainty ratings were assessed conservatively. For the association between PCs and overall systemic diseases, the certainty of evidence was rated as low. Although the pooled association showed a large effect estimate (OR = 2.69), no upgrade was applied because of residual confounding, risk of bias, and methodological heterogeneity. Substantial heterogeneity was observed; however, the direction of effect was consistent across all 22 studies, and leave‐one‐out sensitivity analysis supported the stability of the pooled estimate. The certainty of evidence for DM and atherosclerosis was also rated as low. These subgroups showed large or very large effect estimates, with no statistical heterogeneity. Yet, the observational design, limited control of confounding, and potential measurement bias precluded upgrading the certainty of evidence. In contrast, the evidence for general CVD was rated as very low because of risk of bias, heterogeneity, imprecision, and confidence intervals crossing the null value. The renal and genetic/specialised conditions group was also rated as very low, reflecting serious indirectness and imprecision due to the inclusion of only two studies and the combination of clinically heterogeneous entities. The non‐specific and multiple conditions group was rated as very low because of residual confounding, high heterogeneity, and broad clinical grouping. Overall, large effect estimates were not considered sufficient to upgrade the certainty of evidence in the presence of observational designs and methodological limitations. Therefore, the evidence should be interpreted as suggestive of a consistent association, rather than as confirmation of a causal or predictive relationship (Table 5; Table S8).

### 3.7 Summary of Pooled Evidence

PCs appear to represent imaging findings influenced by both systemic susceptibility and local anatomical modulation. Although the pooled evidence showed a significant association between systemic conditions and PCs (OR = 2.69), the disease‐specific subgroup analyses provided the most clinically meaningful findings. The most consistent association was observed for DM (OR = 2.30; *I* ^2^ = 0.0%), whereas the strongest association was identified in the atherosclerosis‐focused subgroup (OR = 4.70; *I* ^2^ = 0.0%). These subgroups also demonstrated statistical consistency, supporting greater biological interpretability than the overall pooled estimate. These results suggest that metabolic and vascular conditions may be associated with mineralising responses within the dental pulp, while the predilection for molars reinforces the role of local anatomical factors, such as pulpal volume and vascular complexity. Conversely, general CVD was not significantly associated with PCs (*p* = 0.155), indicating that broad cardiovascular classification is less informative than specific atherosclerotic processes. Findings from the renal and genetic/specialised conditions subgroup were interpreted separately and only as exploratory, because this subgroup was based on two studies and combined clinically heterogeneous conditions. Although imaging‐related limitations contributed to variability in prevalence estimates, leave‐one‐out analyses confirmed the stability of the pooled findings. Overall, PCs should be interpreted as associated imaging findings within a multifactorial framework, rather than causal, early, predictive, or diagnostic indicators of patient‐specific systemic risk.

## 4 Discussion

The integration of both quantitative and qualitative data from 19 057 patients supports the interpretation of the dental pulp as a sensitive microenvironment potentially associated with systemic disturbances (Table 3). This challenges the traditional paradigm of PC as a merely local, age‐dependent degenerative event. This study integrates fragmented evidence and demonstrates a significant global association (OR = 2.69). This pooled estimate should be interpreted cautiously, as it combines systemic conditions with distinct pathophysiological profiles (Table 4; Figure 6). Greater clinical and biological interpretability is provided by the disease‐specific subgroup analyses, particularly those focused on DM and atherosclerosis‐related conditions. The in‐depth analysis suggests that PC may not be entirely random, but rather may occur in association with specific metabolic and vascular conditions (Figure 5).

**TABLE 4:** Quantitative outcome data extracted for meta‐analysis from included studies.

Study	Eligibility	Reason for exclusion	Experimental events (*n*)	Experimental total (*N*)	Control events (*n*)	Control total (*N*)
Näsström et al. (1985)	Excluded	No healthy control group; internal comparison only (transplant vs. non‐uraemic vs. dialysis)	—	—	—	—
Moura and Paiva (1987)	Included	—	23	25	10	25
Edds et al. (2005)	Included	—	14	19	14	36
Bauss et al. (2008)	Included	—	15	21	48	100
Gulsahi et al. (2009)	Excluded	Aggregate prevalence reported; no extractable patient‐level *n*/*N* data	—	—	—	—
Horsley et al. (2009)	Included	—	29	61	51	186
Kansu et al. (2009)	Excluded	No healthy control group; comparison limited to renal subgroups	—	—	—	—
Şener et al. (2009)	Included	—	25	56	151	411
Nayak et al. (2010)	Included	—	67	90	15	30
Ezoddini‐Ardakani et al. (2011)	Included	—	31	38	11	23
Kanjanabuch et al. (2011)	Excluded	Outcomes reported as continuous mean percentages; no dichotomous data available	—	—	—	—
Vidyullatha et al. (2011)	Excluded	Descriptive study without a control group	—	—	—	—
Khojastepour et al. (2013)	Included	—	30	44	22	78
Patil and Sinha (2013)	Excluded	All participants affected by ESRD; no control arm included	—	—	—	—
Bains et al. (2014)	Excluded	No distinct healthy control group; intra‐disease comparison only	—	—	—	—
Özakar İlday et al. (2014)	Excluded	‘No disease’ group is not defined as a true healthy control	—	—	—	—
Talla et al. (2014)	Included	—	114	311	404	1689
Çağlayan et al. (2015)	Excluded	Continuous outcomes (mean number of teeth); no patient‐level dichotomous counts	—	—	—	—
Swathy et al. (2015)	Included	—	13	30	12	30
Alsweed et al. (2019)	Included	—	2	41	91	1972
Mathew et al. (2019)	Included	—	433	502	247	528
Simsek et al. (2019)	Included	—	5	83	6	93
Gunen Yilmaz et al. (2019)	Excluded	Internal comparison within haemodialysis cohort only	—	—	—	—
Araya et al. (2020)	Included	—	11	22	6	23
Srivastava et al. (2020)	Included	—	42	149	9	80
Jawahar et al. (2021)	Included	—	18	22	12	48
Ozsin Ozler et al. (2022)	Excluded	No control group included.	—	—	—	—
Alamoudi et al. (2023)	Included	—	26	43	74	157
Kaabi et al. (2024)	Included	—	39	106	117	538
de Souza Romano et al. (2024)	Included	—	41	58	201	432
Beytaş Alğan et al. (2025)	Included	—	1231	2050	783	2000
Baghestani et al. (2025)	Included	—	41	107	64	300
Fairozekhan et al. (2025)	Included	—	26	46	40	107

The association between DM and PCs emerged as the most consistent finding in this review, distinguished by a complete absence of statistical heterogeneity (*I* ^2^ = 0.0%) (Tables 4, 5; Figure 7). The remarkable stability across distinct populations suggests that chronic hyperglycaemia may represent a systemic metabolic condition consistently associated with ectopic mineralisation. This is supported by evidence that high‐glucose environments upregulate the expression of osteopontin (OPN) and alkaline phosphatase within pulp tissue (Araya et al. 2020; Gunen Yilmaz et al. 2019). Consequently, in DM, the pulp may be exposed to a systemic metabolic environment that favours mineralising responses. The pooled OR (2.30) indicates a consistent association between DM and PCs. Biologically, this association may reflect the influence of systemic oxidative stress and altered calcium–phosphate homeostasis on pulpal mineralisation. However, it should still be interpreted as an observational association, since the available studies do not allow the directionality of this relationship to be established.

**TABLE 5:** GRADE summary of evidence for the association between pulp calcification and systemic diseases.

Outcomes	Studies (*n*)	Design	Risk of bias	Inconsistency	Indirectness	Imprecision	Publication bias	Effect magnitude	Certainty
Pulp calcification and overall systemic diseases	22	Observational	Serious	Not serious	Not serious	Not serious	Not serious	Large	LOW ⊕ ⊕ ◯◯
Pulp calcification and diabetes mellitus	3	Observational	Serious	Not serious	Not serious	Serious	Not serious	Large	LOW ⊕ ⊕ ◯◯
Pulp calcification and atherosclerosis	5	Observational	Serious	Not serious	Not serious	Not serious	Not serious	Very large	LOW ⊕ ⊕ ◯◯
Pulp calcification and cardiovascular diseases	5	Observational	Serious	Serious	Not serious	Serious	Not serious	Moderate	VERY LOW ⊕◯◯◯
Pulp calcification and specialised conditions (renal or genetic)	2	Observational	Serious	Not serious	Serious	Very serious	Not assessed	Large	VERY LOW ⊕◯◯◯
Pulp calcification and non‐specific/multiple conditions	7	Observational	Serious	Serious	Serious	Not serious	Not serious	Large	VERY LOW ⊕◯◯◯

Concurrently, the atherosclerosis‐focused analysis presented the highest magnitude of association (OR = 4.70), supporting the hypothesis that pulpal calcification may share biological pathways with vascular calcification. While general CVD did not reach statistical significance (*p* = 0.155), the isolation of specific atherosclerotic markers revealed a distinct connection (Figure 7). This dichotomy suggests that the association becomes more coherent when the exposure is specifically related to atherosclerosis or vascular calcification. Future studies should therefore avoid grouping heterogeneous cardiovascular conditions without mechanistic stratification. In this context, the dental pulp may represent a terminal microcirculatory environment in which systemic vascular alterations are reflected through mineralising changes, although current evidence cannot determine whether these pulpal alterations occur before, during, or after vascular disease progression. The presence of OPN in both carotid plaques and pulp stones (Edds et al. 2005; Baghestani et al. 2025) is compatible with the possibility of overlapping inflammatory pathways involving C‐reactive protein and lipid metabolism dysregulation. Thus, pulpal mineralisation may reflect biological pathways also involved in macrovascular pathology, particularly inflammatory and lipid‐related mechanisms.

The thematic analysis (Braun and Clarke 2006) added interpretative value by organising evidence that could not be incorporated into the meta‐analysis. Rather than functioning as a parallel narrative summary, it clarified how three recurrent dimensions interact: *systemic conditions* may act as biological triggers, *local anatomy* may modulate the expression of calcification, and *imaging limitations* may condition its detection. This framework supported the interpretation of why statistically similar associations may show different prevalence estimates across studies (Figure 5; Table S7). The consistent predilection for molars (Table 3) might be attributed to their larger pulpal volume and complex vascularity, which provide a propitious substrate for mineral deposition (Nayak et al. 2010; Fairozekhan et al. 2025). This suggests a possible interaction in which systemic conditions may contribute to susceptibility, while the anatomical characteristics of molars may modulate the local expression of calcification. Consequently, the observation of PCs may represent the clinical intersection between anatomical vulnerability and systemic susceptibility.

Collectively, these findings support the characterisation of the dental pulp as a tissue potentially responsive to systemic metabolic and vascular disturbances. This concept proposes that the pulp may undergo observable imaging changes in association with chronic metabolic and vascular impairments. Yet, whether these changes precede systemic clinical manifestations remains unknown and requires longitudinal confirmation. Evidence suggests that calcification may occur in systemic contexts not fully explained by physiological ageing. Some studies, such as those involving Marfan syndrome (Bauss et al. 2008) and unerupted teeth (Kaabi et al. 2024), reported calcification patterns in younger cohorts or in teeth with limited exposure to local irritants (Table 3). However, these observations arise from clinically specific and heterogeneous contexts and should not be generalised. At most, they suggest that multiple or apparently idiopathic PCs may represent an imaging pattern compatible with systemic mineralisation tendencies in selected populations, rather than evidence of a broadly applicable systemic mechanism.

The reliability of the primary literature is challenged by significant methodological limitations identified in this review (Figures 3, 4; Tables S4–S6), which necessitate a cautious interpretation of the magnitude of effect. The most relevant concern was the insufficient control of confounding, since age, dental status, restorations, trauma, smoking, medication use, diet, and systemic comorbidity burden may influence both the occurrence of PCs and the probability of systemic disease. Unequal distribution of these variables may inflate the observed OR by attributing calcification to systemic disease rather than shared background risk factors, whereas controls with undiagnosed or related conditions may attenuate the association. A critical assessment also reveals that historical associations may have been distorted by ‘sick control’ bias. For instance, Moura and Paiva (1987) utilised control groups comprising patients with non‐obstructive cardiopathies rather than strictly healthy individuals, which may have diluted differences between groups and underestimated the association. Conversely, in studies with limited adjustment for local, behavioural, pharmacological, or dietary factors, the observed OR may have been overestimated. The impact of this bias may also have differed across subgroups: it may have reduced contrast in the general CVD subgroup, while the atherosclerosis‐focused subgroup remained more biologically coherent because the exposure was closer to vascular calcification. For DM, the absence of heterogeneity suggests consistency, although residual confounding by age, medication use, diet, dental status, and comorbidity burden cannot be excluded. These considerations were incorporated into the GRADE assessment and contributed to conservative downgrading across outcomes. Furthermore, internal inconsistencies in data reporting, such as the numerical discrepancies observed between methodology and results in Patil and Sinha (2013), underscore the fragility of older evidence and justify the strict eligibility criteria applied in our meta‐analysis (Table S3). Thus, the pooled estimate indicates a consistent association, but should not be interpreted as a precise measure of causal effect.

A notable dichotomy exists between the high biological plausibility of these associations and the variability in prevalence estimates. The predominant reliance on bidimensional imaging constitutes a major diagnostic constraint (Table 3). The literature consistently confirms that periapical and panoramic radiographs fail to detect calcifications smaller than 200 μm due to resolution limits and structural superimposition (Bauss et al. 2008; Gulsahi et al. 2009). This implies that the associations reported here likely represent a conservative approximation of a more prevalent histological reality (Table 4; Figure 7). Periapical and panoramic radiographs cannot reliably detect smaller or overlapping calcifications; thus, false‐negative classification may occur and attenuate the observed associations. This limitation may also partly explain the variability in prevalence estimates across studies.

Despite these strengths, this review is subject to limitations inherent to the available data. Patient‐level data were prioritised to reduce clustering bias and avoid artificial inflation of sample size from tooth‐level observations. Nonetheless, this conservative approach limited the detailed assessment of intraoral distribution. Anatomical patterns, particularly molar predilection, were accordingly interpreted mainly through the narrative and thematic analysis (Table 3). This limitation was incorporated into the GRADE assessment of the certainty of evidence. Nevertheless, the leave‐one‐out sensitivity analyses confirmed the robustness of the global estimates (Table 5), mitigating concerns regarding single‐study dominance. Additionally, the absence of significant publication bias supports the reliability of the pooled effect estimates (Figure 8), albeit this finding should be interpreted with caution given the limited number of included studies. By following Braun and Clarke's (2006) framework, the thematic analysis allowed the entire evidence base to be incorporated into the synthesis, including studies that were not eligible for meta‐analysis (Table 4). This approach identified overarching conceptual patterns not fully captured by the quantitative analysis, transforming qualitative findings into structured evidence relevant to the clinical discussion (Table S7). In contrast to the limitations, this review presents significant methodological strengths. This strategic mixed‐methods approach went beyond statistical pooling and delivered a conservative, reproducible, and comprehensive evidence base. Biological plausibility and clinical reality were rigorously integrated; relevant evidence was not disregarded because it was not amenable to quantitative meta‐analysis.

Finally, the clinical implications of imaging‐detected PC should be interpreted with caution. Although PCs were statistically associated with systemic metabolic and vascular conditions, the available evidence does not provide diagnostic accuracy data, such as sensitivity, specificity, or predictive values; therefore, PC cannot currently be used to diagnose systemic disease, estimate individual risk, or guide clinical decision‐making in isolation. Clinically, isolated calcifications in teeth with extensive restorations, caries, trauma, periodontal compromise, occlusal overload, or advanced age should primarily be interpreted within a local or degenerative framework. In contrast, multiple, bilateral, extensive, or apparently idiopathic calcifications, especially when disproportionate to age or present in teeth without evident local irritants, may justify a broader review of the patient's medical history and systemic background. Even so, PC should be considered an associated imaging finding, not a diagnostic marker. The predominance of cross‐sectional designs also prevents determination of whether systemic disease precedes PC formation, develops concurrently with it, or shares common background risk factors. Moreover, global generalisability remains limited by regional differences in dietary habits, genetic background, access to dental care, imaging protocols, and systemic disease profiles. Future longitudinal studies using high‐resolution CBCT are needed to clarify temporal sequence, assess diagnostic accuracy, and determine whether PC has any valid role in systemic risk assessment within integrated healthcare.

## 5 Conclusion

Current evidence supports a positive and statistically significant association between imaging‐detected PCs and a range of systemic conditions. The most clinically meaningful and biologically interpretable findings were observed in the disease‐specific subgroup analyses, particularly for DM and atherosclerosis‐related conditions. Although the overall pooled estimate indicated approximately threefold higher odds of occurrence among exposed individuals, this finding should be interpreted with caution, as disease‐specific analyses provided the most relevant clinical and biological insights. However, predominantly observational studies support this association; thus, it should not be interpreted as causal, predictive, or diagnostic. PC exhibits low specificity for isolated pathologies and is influenced by local anatomical modulation, but it may represent an imaging finding associated with systemic metabolic or vascular dysregulation in selected contexts. Clinicians should interpret PC in conjunction with medical history, dental status, age, and local risk factors. These findings expand the interpretative scope of dental imaging, but they do not currently justify the use of pulpal mineralisation as an opportunistic screening tool or independent marker of systemic risk. Future longitudinal studies using high‐resolution imaging are needed to clarify temporal relationships, assess diagnostic accuracy, and determine whether the pulp‐dentine complex has any valid role in systemic risk stratification.

## Author Contributions

**José Evando da Silva‐Filho:** conceptualisation; data curation; formal analysis; investigation; methodology; writing – original draft; writing – review and editing. **Paula Maria da Costa Torres:** data curation; methodology; supervision; writing – review and editing. **Eduardo Diogo Gurgel‐Filho:** conceptualisation; data curation; methodology; project administration; supervision; writing – review and editing.

## Funding

The authors have nothing to report.

## Disclosure

**Protocol:** José Evando da Silva‐Filho, Paula Maria da Costa Torres, Eduardo Diogo Gurgel‐Filho. Pulp calcification as an imaging biomarker of systemic disease: systematic review, thematic analysis, and meta‐analysis. PROSPERO 2025 CRD420251241536. Available from https://www.crd.york.ac.uk/PROSPERO/view/CRD420251241536.

## Conflicts of Interest

The authors declare no conflicts of interest.

## Supporting information


**Figure S1:** Leave‐one‐out sensitivity analysis forest plot showing the stability of the pooled effect estimate regarding the association between systemic conditions and pulp calcification.

**Figure S2:** Leave‐one‐out sensitivity analysis forest plot showing the stability of the pooled effect estimate regarding the association between diabetes mellitus and pulp calcification.

**Figure S3:** Leave‐one‐out sensitivity analysis forest plot showing the stability of the pooled effect estimate regarding the association between general cardiovascular diseases and pulp calcification.

**Figure S4:** Leave‐one‐out sensitivity analysis forest plot showing the stability of the pooled effect estimate regarding the association between atherosclerosis‐related conditions and pulp calcification.

**Figure S5:** Leave‐one‐out sensitivity analysis forest plot showing the stability of the pooled effect estimate regarding the association between non‐specific and multiple systemic conditions and pulp calcification.

**Table S1:** Full search strategy for each database.
**Table S2:** Additional studies identified through references list checking.
**Table S3:** Reasons for exclusion of studies after full‐text evaluation.
**Table S4:** Methodological appraisal of included studies using the Joanna Briggs Institute (JBI) Critical Appraisal Checklist.
**Table S5:** Methodological quality assessment of included case–control studies using the Newcastle–Ottawa Scale (NOS). Studies were evaluated across three domains: selection, comparability, and exposure. Overall methodological quality was categorised as high (7–9 stars), moderate (4–6 stars), or low (0–3 stars).
**Table S6:** Methodological quality assessment of included cohort studies using the Newcastle–Ottawa Scale (NOS). Studies were evaluated across three domains: selection, comparability, and outcome. Overall methodological quality was categorised as high (7–9 stars), moderate (4–6 stars), or low (0–3 stars).
**Table S7:** Stepwise thematic synthesis of findings across the included studies (*n* = 33).
**Table S8:** GRADE justification for certainty of evidence assessments.

## Data Availability

The data that supports the findings of this study are available in the Supporting Information of this article.
