# Supplementary material for: Associations Between Imaging‐Detected Pulp Calcification and Systemic Diseases: A Systematic Review, Thematic Analysis, and Meta‐Analysis
Source: Int Endod J. 2026 Jul 19;59(11):2283–302. doi: 10.1111/iej.70230 (PMC13674849; doi:10.1111/iej.70230)

Study	OR	95%−CI	P−value	Tau2	Tau	I2
Omitting Nayak et al., 2010	2.37	[1.21; 4.64]	0.0216	0.3366	0.5801	91.1%
Omitting Talla et al., 2014	2.57	[1.30; 5.08]	0.0163	0.3311	0.5755	89.9%
Omitting Mathew et al., 2019	2.11	[1.75; 2.56]	0.0002	0.0092	0.0958	10.6%
Omitting Simsek et al., 2019	2.65	[1.50; 4.69]	0.0071	0.2655	0.5153	90.7%
Omitting Alamoudi et al., 2023	2.56	[1.32; 4.97]	0.0148	0.3180	0.5639	90.9%
Omitting Kaabi et al., 2024	2.49	[1.25; 4.96]	0.0194	0.3484	0.5903	91.0%
Omitting Algan et al., 2025	2.43	[1.21; 4.92]	0.0226	0.3654	0.6045	90.5%
**Random effects model**	**2.44**	**[1.41; 4.24]**	**0.0075**	**0.2838**	**0.5327**	**89.3%**

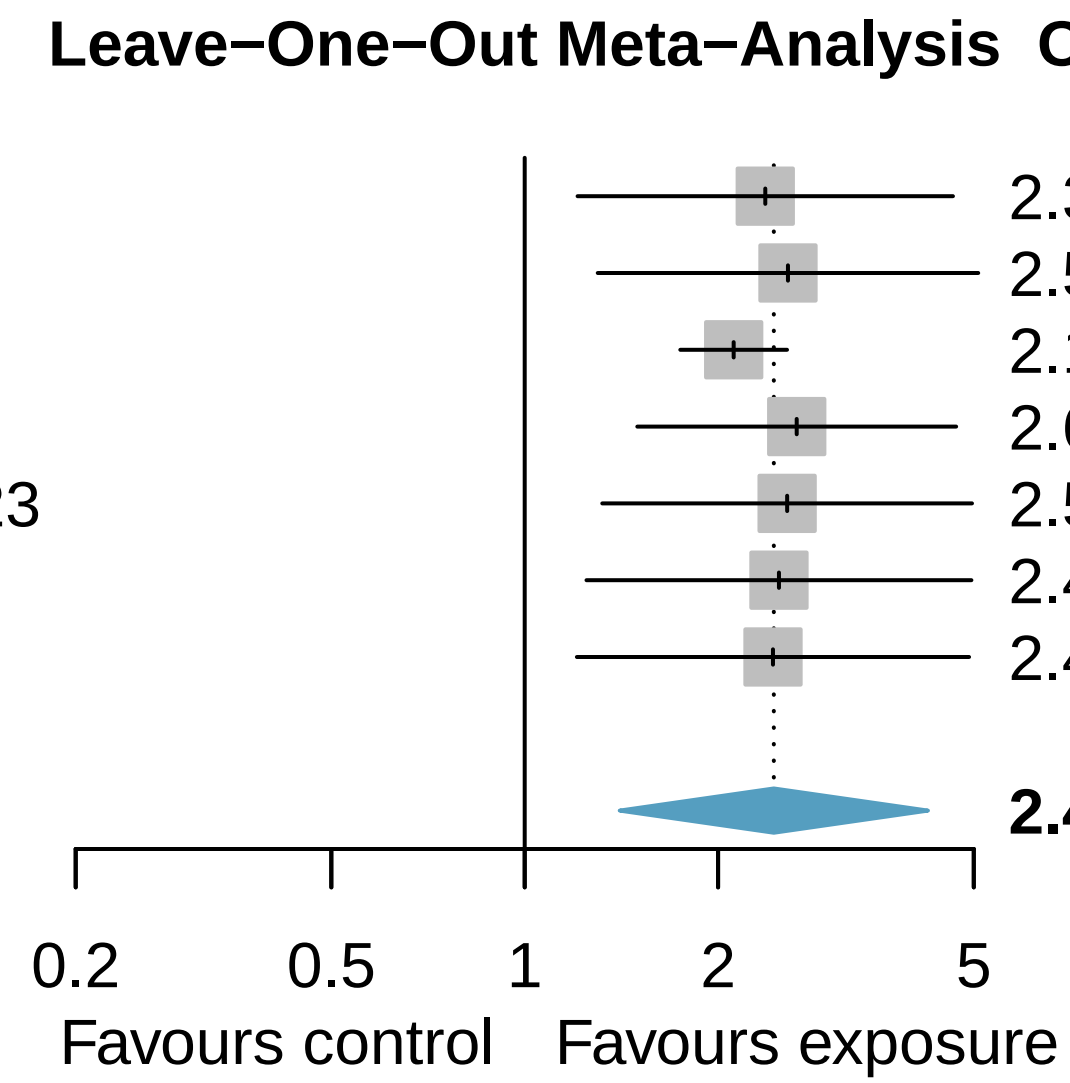

Supplement: Supplementary file 1 [file IEJ-59-2283-s001.pdf]
