# Supplementary material for: Associations Between Imaging‐Detected Pulp Calcification and Systemic Diseases: A Systematic Review, Thematic Analysis, and Meta‐Analysis
Source: Int Endod J. 2026 Jul 19;59(11):2283–302. doi: 10.1111/iej.70230 (PMC13674849; doi:10.1111/iej.70230)
Supplement: Supplementary file 2 [file IEJ-59-2283-s002.pdf]

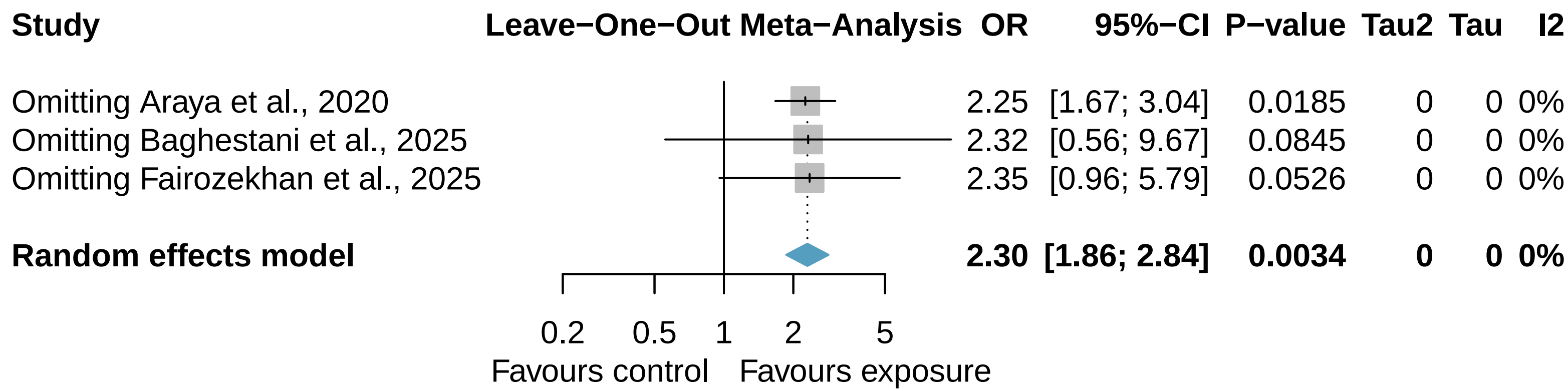
Study
Leave−One−Out Meta−Analysis
OR
95%−CI
P−value
Tau2
Tau
I2
Omitting Araya et al., 2020 2.25 [1.67; 3.04] 0.0185 0 0 0%
Omitting Baghestani et al., 2025 2.32 [0.56; 9.67] 0.0845 0 0 0%
Omitting Fairozekhan et al., 2025 2.35 [0.96; 5.79] 0.0526 0 0 0%
Random effects model 2.30 [1.86; 2.84] 0.0034 0 0 0%
0.2
0.5
1
2
5
Favours control
Favours exposure
