# Supplementary material for: Associations Between Imaging‐Detected Pulp Calcification and Systemic Diseases: A Systematic Review, Thematic Analysis, and Meta‐Analysis
Source: Int Endod J. 2026 Jul 19;59(11):2283–302. doi: 10.1111/iej.70230 (PMC13674849; doi:10.1111/iej.70230)

Study	Leave−One−Out Meta−Analysis	OR	95%−CI	P−value	Tau2	Tau	I2
Omitting Moura et al., 1987		2.60	[2.00; 3.37]	< 0.0001	0.1745	0.4177	74.1%
Omitting Edds et al., 2005		2.65	[1.99; 3.52]	< 0.0001	0.1953	0.4420	75.5%
Omitting Bauss et al., 2008		2.69	[2.01; 3.59]	< 0.0001	0.2027	0.4502	75.7%
Omitting Horsley et al., 2009		2.71	[2.02; 3.63]	< 0.0001	0.2097	0.4579	75.7%
Omitting Sener et al., 2009		2.79	[2.11; 3.69]	< 0.0001	0.1791	0.4232	74.3%
Omitting Nayak et al., 2010		2.68	[2.00; 3.58]	< 0.0001	0.2048	0.4525	75.7%
Omitting Ezoddini et al., 2011		2.64	[1.99; 3.50]	< 0.0001	0.1933	0.4396	75.3%
Omitting Khojastepour et al., 2013		2.60	[1.96; 3.43]	< 0.0001	0.1821	0.4268	74.6%
Omitting Talla et al., 2014		2.77	[2.07; 3.71]	< 0.0001	0.1985	0.4456	73.6%
Omitting Swathi et al., 2015		2.77	[2.10; 3.65]	< 0.0001	0.1862	0.4315	75.0%
Omitting Alsweed et al., 2019		2.74	[2.07; 3.62]	< 0.0001	0.1908	0.4368	75.3%
Omitting Mathew et al., 2019		2.28	[1.94; 2.68]	< 0.0001	0.0055	0.0743	40.6%
Omitting Simsek et al., 2019		2.76	[2.10; 3.63]	< 0.0001	0.1844	0.4294	74.9%
Omitting Araya et al., 2020		2.68	[2.01; 3.58]	< 0.0001	0.1998	0.4470	75.7%
Omitting Srivastava et al., 2020		2.67	[2.00; 3.57]	< 0.0001	0.2052	0.4530	75.6%
Omitting Jawahar et al., 2021		2.57	[1.99; 3.32]	< 0.0001	0.1639	0.4048	73.5%
Omitting Alamoudi et al., 2023		2.75	[2.07; 3.67]	< 0.0001	0.1978	0.4448	75.3%
Omitting Kaabi et al., 2024		2.74	[2.04; 3.67]	< 0.0001	0.2081	0.4562	75.5%
Omitting Romano et al., 2024		2.69	[2.00; 3.60]	< 0.0001	0.2101	0.4583	75.7%
Omitting Algan et al., 2025		2.72	[2.02; 3.66]	< 0.0001	0.2160	0.4647	74.8%
Omitting Baghestani et al., 2025		2.72	[2.03; 3.65]	< 0.0001	0.2109	0.4593	75.7%
Omitting Fairozekhan et al., 2025		2.72	[2.03; 3.64]	< 0.0001	0.2062	0.4541	75.7%
**Random effects model**		**2.69**	**[2.04; 3.54]**	**< 0.0001**	**0.1906**	**0.4366**	**74.5%**

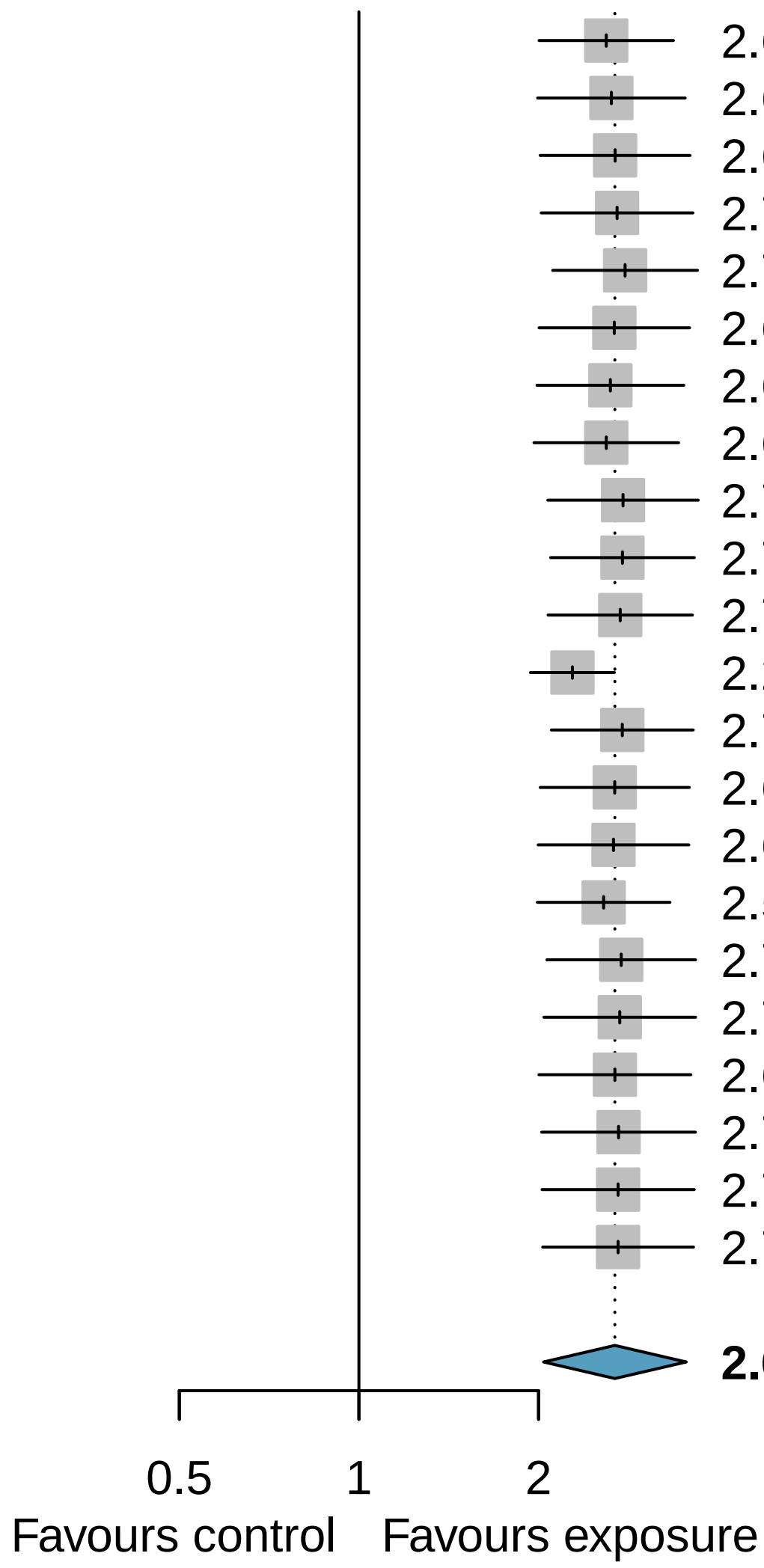

Supplement: Supplementary file 3 [file IEJ-59-2283-s003.pdf]
