# Supplementary material for: Associations Between Imaging‐Detected Pulp Calcification and Systemic Diseases: A Systematic Review, Thematic Analysis, and Meta‐Analysis
Source: Int Endod J. 2026 Jul 19;59(11):2283–302. doi: 10.1111/iej.70230 (PMC13674849; doi:10.1111/iej.70230)
Supplement: Supplementary file 4 [file IEJ-59-2283-s004.pdf]

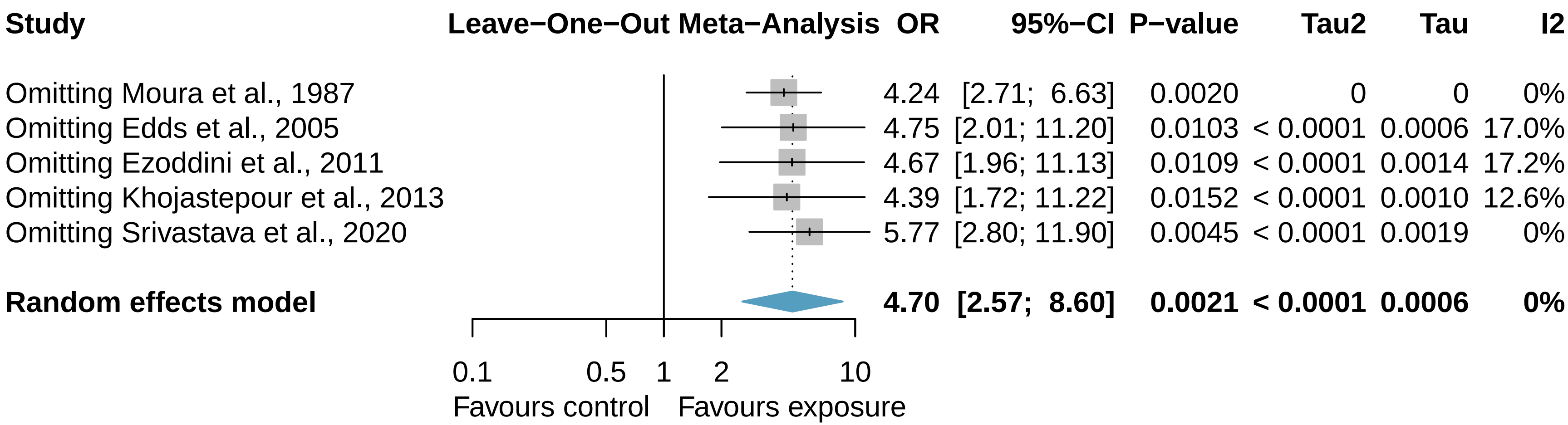
Study
Leave−One−Out Meta−Analysis
OR
95%−CI
P−value
Tau2
Tau
I2
Omitting Moura et al., 1987 4.24 [2.71; 6.63] 0.0020 0 0 0%
Omitting Edds et al., 2005 4.75 [2.01; 11.20] 0.0103 < 0.0001 0.0006 17.0%
Omitting Ezoddini et al., 2011 4.67 [1.96; 11.13] 0.0109 < 0.0001 0.0014 17.2%
Omitting Khojastepour et al., 2013 4.39 [1.72; 11.22] 0.0152 < 0.0001 0.0010 12.6%
Omitting Srivastava et al., 2020 5.77 [2.80; 11.90] 0.0045 < 0.0001 0.0019 0%
Random effects model 4.70 [2.57; 8.60] 0.0021 < 0.0001 0.0006 0%
0.1
0.5
1
2
10
Favours control
Favours exposure
