# Supplementary material for: Associations Between Imaging‐Detected Pulp Calcification and Systemic Diseases: A Systematic Review, Thematic Analysis, and Meta‐Analysis
Source: Int Endod J. 2026 Jul 19;59(11):2283–302. doi: 10.1111/iej.70230 (PMC13674849; doi:10.1111/iej.70230)
Supplement: Supplementary file 5 [file IEJ-59-2283-s005.pdf]

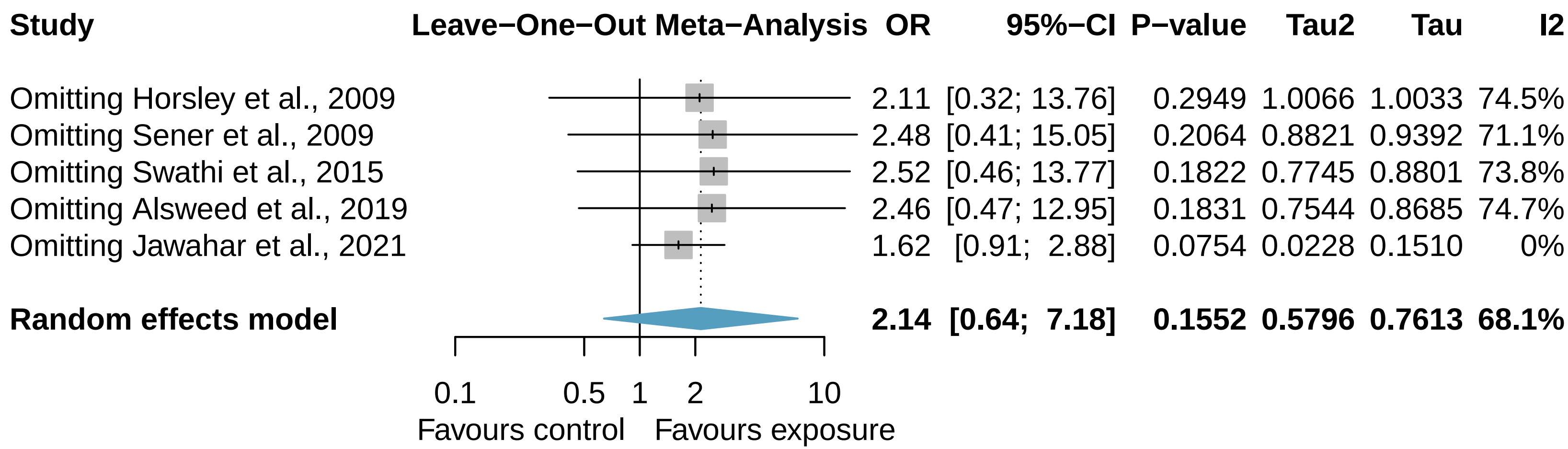
Study
Leave−One−Out Meta−Analysis
OR
95%−CI
P−value
Tau2
Tau
I2
Omitting Horsley et al., 2009 2.11 [0.32; 13.76] 0.2949 1.0066 1.0033 74.5%
Omitting Sener et al., 2009 2.48 [0.41; 15.05] 0.2064 0.8821 0.9392 71.1%
Omitting Swathi et al., 2015 2.52 [0.46; 13.77] 0.1822 0.7745 0.8801 73.8%
Omitting Alsweed et al., 2019 2.46 [0.47; 12.95] 0.1831 0.7544 0.8685 74.7%
Omitting Jawahar et al., 2021 1.62 [0.91; 2.88] 0.0754 0.0228 0.1510 0%
Random effects model 2.14 [0.64; 7.18] 0.1552 0.5796 0.7613 68.1%
0.1
0.5
1
2
10
Favours control
Favours exposure
