# Supplementary material for: Associations Between Imaging‐Detected Pulp Calcification and Systemic Diseases: A Systematic Review, Thematic Analysis, and Meta‐Analysis
Source: Int Endod J. 2026 Jul 19;59(11):2283–302. doi: 10.1111/iej.70230 (PMC13674849; doi:10.1111/iej.70230)
Supplement: Supplementary file 6 [file IEJ-59-2283-s006.docx]

SUPPLEMENTARY TABLES

**Supplementary Table S1.** Full search strategy for each database.

| **Database** | **Strategy** |
| --- | --- |
| Embase | ('dental pulp calcification':ti,ab,kw OR 'pulp calcification':ti,ab,kw OR 'dental pulp stone*':ti,ab,kw OR 'pulp stone*':ti,ab,kw) AND ('diagnostic imaging':ti,ab,kw OR 'radiography':ti,ab,kw OR 'dental radiography':ti,ab,kw OR 'dental radiograph*':ti,ab,kw OR cbct:ti,ab,kw OR 'cone beam computed tomography':ti,ab,kw OR 'panoramic radiograph*':ti,ab,kw) AND ('systemic disease*':ti,ab,kw OR 'syndrome*':ti,ab,kw OR 'genetic disorder*':ti,ab,kw OR 'endocrine disorder*':ti,ab,kw OR 'metabolic disorder*':ti,ab,kw OR 'autoimmune disease*':ti,ab,kw OR 'inflammatory disease*':ti,ab,kw OR 'cardiovascular disease*':ti,ab,kw OR 'hypertension':ti,ab,kw OR 'hyperlipidemia':ti,ab,kw OR 'atherosclerosis':ti,ab,kw OR 'diabetes mellitus':ti,ab,kw OR 'obesity':ti,ab,kw OR 'renal disease*':ti,ab,kw OR 'chronic kidney disease*':ti,ab,kw OR 'hepatic disease*':ti,ab,kw OR 'respiratory disease*':ti,ab,kw) |
| ProQuest (Dissertations & Thesis) | ("dental pulp calcification" OR "pulp calcification" OR "dental pulp stone" OR "pulp stone") AND ("diagnostic imaging" OR "radiography" OR "dental radiography" OR "dental radiograph" OR CBCT OR "cone beam computed tomography" OR "panoramic radiograph") AND ("systemic disease" OR "syndrome" OR "genetic disorder" OR "endocrine disorder" OR "metabolic disorder" OR "autoimmune disease" OR "inflammatory disease" OR "cardiovascular disease" OR "hypertension" OR "hyperlipidemia" OR "atherosclerosis" OR "diabetes mellitus" OR "obesity" OR "renal disease" OR "chronic kidney disease" OR "hepatic disease" OR "respiratory disease") |
|  | ("dental pulp calcification" OR "pulp calcification" OR "dental pulp stone" OR "pulp stone") AND ("diagnostic imaging" OR radiography OR CBCT OR "cone beam computed tomography" OR "panoramic radiograph") AND ("systemic disease" OR syndrome OR "genetic disorder" OR "endocrine disorder" OR "metabolic disorder" OR "autoimmune disease" OR "inflammatory disease" OR "cardiovascular disease" OR diabetes OR obesity OR "renal disease" OR "hepatic disease" OR "respiratory disease") |
| PubMed | ("Dental Pulp Calcification"[Mesh] OR "Dental Pulp Calcification"[tiab] OR "Dental Pulp Calcifications"[tiab] OR "Pulp Calcification"[tiab] OR "Pulp Calcifications"[tiab] OR "Dental Pulp Stone*"[tiab] OR "Pulp Stone*"[tiab] OR "Dental Pulp Stones"[tiab] OR "Pulp Stones"[tiab]) AND ("Systemic Disease*"[tiab] OR "Syndrome*"[tiab] OR "Genetic Disorder*"[tiab] OR "Endocrine Disorder*"[tiab] OR "Metabolic Disorder*"[tiab] OR "Autoimmune Disease*"[tiab] OR "Inflammatory Disease*"[tiab] OR "Cardiovascular Disease*"[tiab] OR "Hypertension"[tiab] OR "Hyperlipidemia"[tiab] OR "Atherosclerosis"[tiab] OR "Diabetes Mellitus"[tiab] OR "Obesity"[tiab] OR "Renal Disease*"[tiab] OR "Chronic Kidney Disease*"[tiab] OR "Hepatic Disease*"[tiab] OR "Respiratory Disease*"[tiab]) |
| SciELO | (pulp calcification OR pulp stone OR dental pulp stone OR dental pulp calcification OR calcificação pulpar OR cálculo pulpar) AND (diagnostic imaging OR radiography OR dental radiography OR panoramic radiograph OR radiografia OR radiografia dental OR radiografia panorâmica OR CBCT OR cone beam computed tomography OR tomografia computadorizada de feixe cônico) AND (systemic disease OR syndrome OR genetic disorder OR endocrine disorder OR metabolic disorder OR autoimmune disease OR inflammatory disease OR cardiovascular disease OR hypertension OR hyperlipidemia OR atherosclerosis OR diabetes mellitus OR obesity OR renal disease OR chronic kidney disease OR hepatic disease OR respiratory disease OR doença sistêmica OR síndrome OR distúrbio genético OR distúrbio endócrino OR distúrbio metabólico OR doença autoimune OR doença inflamatória OR doença cardiovascular OR hipertensão OR hiperlipidemia OR aterosclerose OR diabetes mellitus OR obesidade OR doença renal OR doença renal crônica OR doença hepática OR doença respiratória) |
|  | (pulp calcification OR pulp stone OR calcificação pulpar) AND (radiografia dental OR radiografia panorâmica OR CBCT OR cone beam)  AND (systemic disease OR doença sistêmica OR diabetes mellitus OR hipertensão OR doença cardiovascular OR doença renal OR obesidade) |
| Scopus | (TITLE-ABS-KEY("Dental Pulp Calcification") OR TITLE-ABS-KEY("Dental Pulp Calcifications") OR TITLE-ABS-KEY("Pulp Calcification") OR TITLE-ABS-KEY("Pulp Calcifications") OR TITLE-ABS-KEY("Dental Pulp Stone") OR TITLE-ABS-KEY("Pulp Stone*") OR TITLE-ABS-KEY("Dental Pulp Stones") OR TITLE-ABS-KEY("Pulp Stones")) AND (TITLE-ABS-KEY("Diagnostic Imaging") OR TITLE-ABS-KEY("Radiography") OR TITLE-ABS-KEY("Dental Radiography") OR TITLE-ABS-KEY("Dental Radiograph*") OR TITLE-ABS-KEY("CBCT") OR TITLE-ABS-KEY("Cone Beam Computed Tomography") OR TITLE-ABS-KEY("Panoramic Radiograph*")) AND (TITLE-ABS-KEY("Systemic Disease*") OR TITLE-ABS-KEY("Syndrome*") OR TITLE-ABS-KEY("Genetic Disorder*") OR TITLE-ABS-KEY("Endocrine Disorder*") OR TITLE-ABS-KEY("Metabolic Disorder*") OR TITLE-ABS-KEY("Autoimmune Disease*") OR TITLE-ABS-KEY("Inflammatory Disease*") OR TITLE-ABS-KEY("Cardiovascular Disease*") OR TITLE-ABS-KEY("Hypertension") OR TITLE-ABS-KEY("Hyperlipidemia") OR TITLE-ABS-KEY("Atherosclerosis") OR TITLE-ABS-KEY("Diabetes Mellitus") OR TITLE-ABS-KEY("Obesity") OR TITLE-ABS-KEY("Renal Disease*") OR TITLE-ABS-KEY("Chronic Kidney Disease*") OR TITLE-ABS-KEY("Hepatic Disease*") OR TITLE-ABS-KEY("Respiratory Disease*")) |
| VHL  (Complete Collection) | ("pulp calcification" OR "pulp stone" OR "dental pulp stone" OR "dental pulp calcification") AND ("diagnostic imaging" OR "radiography" OR "dental radiography" OR "panoramic radiograph" OR CBCT OR "cone beam computed tomography") AND ("systemic disease" OR "syndrome" OR "genetic disorder" OR "endocrine disorder" OR "metabolic disorder" OR "autoimmune disease" OR "inflammatory disease" OR "cardiovascular disease" OR "hypertension" OR "hyperlipidemia" OR "atherosclerosis" OR "diabetes mellitus" OR "obesity" OR "renal disease" OR "chronic kidney disease" OR "hepatic disease" OR "respiratory disease") |
|  | ("calcificação pulpar" OR "cálculo pulpar" OR "pulp calcification" OR "pulp stone" OR "dental pulp stone" OR "dental pulp calcification") AND ("imagem diagnóstica" OR "radiografia" OR "radiografia dental" OR "radiografia panorâmica" OR CBCT OR "cone beam computed tomography") AND ("doença sistêmica" OR "síndrome" OR "distúrbio genético" OR "distúrbio endócrino" OR "distúrbio metabólico" OR "doença autoimune" OR "doença inflamatória" OR "doença cardiovascular" OR "hipertensão" OR "hiperlipidemia" OR "aterosclerose" OR "diabetes mellitus" OR "obesidade" OR "doença renal" OR "doença renal crônica" OR "doença hepática" OR "doença respiratória") |
| Web of Science | TS=("Dental Pulp Calcification" OR "Dental Pulp Calcifications" OR "Pulp Calcification" OR "Pulp Calcifications" OR "Dental Pulp Stone*" OR "Pulp Stone*" OR "Dental Pulp Stones" OR "Pulp Stones") AND TS=("Diagnostic Imaging" OR "Radiography" OR "Dental Radiography" OR "Dental Radiograph*" OR CBCT OR "Cone Beam Computed Tomography" OR "Panoramic Radiograph*") AND TS=("Systemic Disease*" OR "Syndrome*" OR "Genetic Disorder*" OR "Endocrine Disorder*" OR "Metabolic Disorder*" OR "Autoimmune Disease*" OR "Inflammatory Disease*" OR "Cardiovascular Disease*" OR "Hypertension" OR "Hyperlipidemia" OR "Atherosclerosis" OR "Diabetes Mellitus" OR "Obesity" OR "Renal Disease*" OR "Chronic Kidney Disease*" OR "Hepatic Disease*" OR "Respiratory Disease*") |

**Supplementary Table S2.** Additional studies identified through references list checking.

| **Title** | **Authors** | **Information** | **Status** |
| --- | --- | --- | --- |
| Narrowing of the dental pulp chamber in patients with renal diseases. | Näsström K, Forsberg B, Petersson A, Westesson P | Oral Surg Oral Med Oral Pathol 1985;59: 242– 6 | Included |
| Prevalence of pulp stones in a teenage Iraqi group. | Baghdady VS, Ghose LJ, Nahoom HY | J Endod 1988;14:309-311 | Not included due to lack of systemic condition assessment |
| Prevalence of pulp stones in Jordanian adults. | Hamasha AA, Darwazeh A | Oral Surg Oral Med Oral Pathol Oral Radiol Endod 1998;86:730-2 | Not included due to lack of systemic condition assessment |
| A radiographic assessment of the prevalence of pulp stones in Australians. | Ranjitkar S, Taylor JA, Townsend GC | Aust Dent J 2002;47:36-40 | Not included due to lack of systemic condition assessment |
| The prevalence of pulp stones in a Turkish population. A radiographic survey. | Sisman Y, Aktan AM, Tarim-Ertas E, Çiftçi ME, Sekerci AE | Med Oral Patol Oral Cir Bucal 2012;17:e212-7 | Not included due to lack of systemic condition assessment. |
| Assessment of the prevalence of pulp stones in a sample of Turkish Central Anatolian population. | Colak H, Celebi AA, Hamidi MM, Bayraktar Y, Colak T, Uzgur R | ScientificWorldJournal 2012;2012:804278 | Not included due to lack of systemic condition assessment |
| Assessing pulp stones by cone-beam computed tomography. | da Silva EJ, Prado MC, Queiroz PM, et al. | Clin Oral Investig 2017;21:2327-33 | Not included due to lack of systemic condition assessment |
| The prevalence of pulp stones in adult patients of Shiraz Dental School, a radiographic assessment. | Ravanshad S, Khayat S, Freidonpour N | J Dent (Shiraz) 2015;16:356-61 | Not included due to lack of systemic condition assessment |

.

**Supplementary Table S3.** Reasons for exclusion of studies after full-text evaluation

| **Study** | **Reason for Exclusion** |
| --- | --- |
| Russell, 1967 | E2 |
| dos Santos-Neto et al., 2011 | E6 |
| de la Dure-Molla et al., 2014 | E2 |
| Sandeepa et al., 2016 | E1 |
| Acharya et al., 2021 | E1 |
| Nicklisch et al., 2021 | E3 |
| Sugiyama et al., 2021 | E5 |
| Hoshyari et al., 2022 | E8 |
| Ozler et al., 2022 | E1 |
| Ishikawa et al., 2023 | E3 |
| Kurosaka et al., 2023 | E4 |
| Khan et al., 2024 | E7 |
| **Exclusion criteria:**  E1. Comparison: no systemic condition data available (control group or exposed group undefined)  E2. Study design: review (not identified by title or abstract)  E3. Population: cadaveric study (incompatible with clinical systemic assessment)  E4. Study design: case report (single observation/lack of control)  E5. Study design: in vitro study;  E6. Outcome not relevant: descriptive analysis tangential to the topic  E7. Study design: histopathological analysis without imaging association  E8. Full-text unavailable | |

**Supplementary Table S4.** Methodological appraisal of included studies using the Joanna Briggs Institute (JBI) Critical Appraisal Checklist.

| **Study** | **Q1** | **Q2** | **Q3** | **Q4** | **Q5** | **Q6** | **Q7** | **Q8** | **Overall risk of bias** |
| --- | --- | --- | --- | --- | --- | --- | --- | --- | --- |
| Moura; Paiva, 1987 | YES – Clearly defined CA and control groups; males aged 40–60 | YES – 50 male patients, University of São Paulo, Brazil | YES – Cardiac diagnosis confirmed by clinical exam, lab tests, and angiocoronariography | YES – Pulpal denticles assessed by long-cone periapical radiographs | UNCLEAR – Other confounders (e.g., smoking, medications) not specified | YES – Age and sex controlled by sample restriction | YES – Standardised radiographic assessment across 570 radiographs | YES – Chi-square test with p ≤ 0.001 | Moderate |
| Edds et al., 2005 | YES – Defined inclusion/exclusion (age 20–55; noncarious molars; no renal/gout history) | YES – 55 dental patients, University of Louisville | UNCLEAR – CVD status self-reported | YES – Blinded periapical radiograph assessment | YES – Key confounders identified (age, restorations, caries, periodontal/renal disease) | YES – Controlled via exclusion criteria | YES – Blinded and verified radiographic evaluation | YES – OR, sensitivity, specificity (95% CI) | Moderate |
| Gulsahi et al., 2009 | YES – Random selection, age 18–54 | YES – Turkish dental patients, n=519 | YES – Full-mouth periapical radiographs, paralleling technique | YES – Presence of radio-opaque masses | UNCLEAR – Confounders not explicitly defined | NO – No strategies stated | YES – Oral radiologist via view box | YES – Chi-square test (SPSS 12.0) | Moderate |
| Şener et al., 2009 | YES – Random selection, age 13–65 | YES – University setting, Turkey, n=536 | YES – Periapical and bitewing radiographs | YES – Definite radiopaque foci | UNCLEAR – Factors mentioned but not defined as confounders | NO – Not reported | YES – Two investigators, κ=0.98 | YES – Chi-square, Spearman correlation | Moderate |
| Nayak et al., 2010 | YES – Defined age and clinical criteria | YES – District population, India, n=150 | YES – RVG and IOPA radiographs | YES – Radiopaque masses within pulp | UNCLEAR – Confounders not identified | NO – Not reported | YES – Viewed under 2× magnification | YES – Chi-square, age regression | Moderate |
| Kanjanabuch et al., 2011 | YES – ESRD vs matched healthy controls | YES – Hospital-based, Thailand, n=30 | YES – Panoramic and bitewing films | YES – Defined pulpal sclerosis/stones | YES – Age and sex identified | YES – Matching strategy | YES – Blinded oral radiologist | YES – t-test, Mann–Whitney U | Low |
| Vidyullatha et al., 2011 | YES – ESRD on hemodialysis | YES – Hospital setting, India, n=40 | YES – Panoramic and periapical radiographs | YES – Standardised grading system | NO – Not reported | NO – Not reported | YES – Two blinded examiners | YES – Descriptive statistics | Moderate |
| Khojastepour et al., 2013 | YES – Defined age and tooth criteria | YES – Angiography department, n=122 | YES – Digital panoramic radiographs | YES – Pulp stones/obliterated chambers | YES – Age and gender assessed | YES – Logistic regression | YES – Blinded radiologist | YES – Sensitivity/specificity analysis | Low |
| Bains et al., 2014 | YES – Age 18–67 with exclusions | YES – Dental outpatients, Punjab, n=500 | YES – Bitewing radiographs with XCP | YES – Radiopaque structure definition | NO – Not reported | NO – Not reported | YES – Viewer and magnification | YES – Chi-square (SPSS 5.0) | Moderate |
| Özakar İlday et al., 2014 | YES – Age 8–72 | YES – Hospital archives, Turkey, n=4798 | YES – Panoramic radiographs and records | YES – Definite radiopaque bodies | NO – Not reported | NO – Not reported | YES – Software magnification, repeat exam | YES – Chi-square (SPSS 16.0) | Moderate |
| Talla et al., 2014 | YES – Age 10–60, systemic groups | YES – Andhra Pradesh, India, n=2000 | YES – Analog IOPA radiographs | YES – Radiopaque masses | NO – Not reported | NO – Not reported | YES – Visual method under safelight | YES – Chi-square (SPSS 15.0) | Moderate |
| Swathy et al., 2015 | YES – Cardiac history vs controls | YES – Dental college/clinic, Chennai, n=60 | YES – IOPA and OPG radiographs | YES – Radiopaque mass in pulp | NO – Not reported | NO – Not reported | YES – Two independent observers | YES – Prevalence analysis | Moderate |
| Simsek et al., 2019 | YES – Age 9–17, BMI criteria | YES – University setting, Turkey, n=186 | YES – Panoramic radiographs | YES – Discrete pulpal calcifications | YES – Age/sex growth charts | YES – BMI categorization | YES – κ=0.915 | YES – Chi-square, Fisher’s exact | Low |
| Jawahar et al., 2021 | YES – Patients undergoing root canal treatment | YES – 70 patients, CSI Dental College, Madurai | YES – HTN/HL diagnosed per AHA 2017 and ACC 2018 | YES – Radiographic and histopathological criteria (H&E) | UNCLEAR – Confounders not clearly specified | NO – No confounder control strategies reported | YES – Standardised pulp tissue processing and microscopy | YES – Chi-square and Student’s t-test | Moderate |
| Ozler et al., 2022 | YES – Pediatric/adolescent IBMFS patients | YES – Hospital setting, Turkey, n=27 | YES – Clinical exam and panoramic radiographs | YES – Clinical/radiological criteria | NO – Not reported | NO – Not reported | YES – Two examiners, κ=0.92 | YES – Descriptive statistics | Moderate |
| **Assessment criteria:**  Q1: Were the criteria for inclusion in the sample clearly defined?  Q2: Were the study subjects and the setting described in detail?  Q3: Was the exposure measured in a valid and reliable way?  Q4: Were objective, standard criteria used for measurement of the condition?  Q5: Were confounding factors identified?  Q6: Were strategies to deal with confounding factors stated?  Q7: Were the outcomes measured in a valid and reliable way?  Q8: Was appropriate statistical analysis used? | | | | | | | | | |

**Supplementary Table S5.** Methodological quality assessment of included case–control studies using the Newcastle–Ottawa Scale (NOS). Studies were evaluated across three domains: selection, comparability, and exposure. Overall methodological quality was categorised as high (7–9 stars), moderate (4–6 stars), or low (0–3 stars).

| **Study** | **Selection**  **(max 4★)** | **Comparability**  **(max 2★)** | **Exposure (max 3★)** | **Total**  **(max 9★)** | **Overall methodological quality** |
| --- | --- | --- | --- | --- | --- |
| Bauss et al., 2008 | ★★☆☆  Cases previously diagnosed with Marfan syndrome; limited representativeness and clinic-based controls | ★★  Matched for age and sex | ★☆☆ Radiographic assessment without explicit blinding; non-response not reported | 5/9 | Moderate |
| Ezoddini-Ardakani et al., 2011 | ★★☆☆ Angiography-confirmed CAD cases; hospital-based controls | ★★  Age and sex controlled (Mantel–Haenszel) | ★★☆ Blinded radiographic assessment; non-response not reported | 6/9 | Moderate |
| Çağlayan et al., 2015 | ★★☆☆ Clinically defined CRF cases from nephrology department; CBCT-archive hospital controls | ★★  Age- and sex-matched; multiple systemic and behavioral confounders excluded | ★★☆  Secure medical records for exposure; identical CBCT protocol; non-response not reported | 6/9 | Moderate |
| Araya et al., 2020 | ★☆☆☆ Convenience sample of DM patients from university clinics | ★☆  Partial control for age only | ★★☆ Calibrated examiners using standardised periapical radiographs | 4/9 | Moderate |
| Srivastava et al., 2020 | ★☆☆☆ Retrospective CBCT-based identification; hospital-based sample | ★★ Multivariable adjustment (age, sex, tooth type) | ★★★ Blinded CBCT assessment; uniform exclusion of poor-quality scans | 6/9 | Moderate |
| Alamoudi et al., 2023 | ★★★☆ Pair-matched case–control design; clinic-based controls | ★★ Pair-matched by age and sex | ★★☆ Medical history obtained for all; participant flow reported | 7/9 | High |
| de Souza-Romano et al., 2024 | ★★☆☆ Radiographically defined cases; hospital-based recruitment | ★★  Groups comparable for age and sex | ★★☆ Standardised questionnaire applied equally; attrition considered | 6/9 | Moderate |
| Kaabi et al., 2024 | ★★☆☆  Large hospital database; clear radiographic definition | ★☆  Limited adjustment for confounders | ★★☆ Uniform CBCT-based data extraction; quality criteria applied | 5/9 | Moderate |

**Supplementary Table S6.** Methodological quality assessment of included cohort studies using the Newcastle–Ottawa Scale (NOS). Studies were evaluated across three domains: selection, comparability, and outcome. Overall methodological quality was categorised as high (7–9 stars), moderate (4–6 stars), or low (0–3 stars).

| **Study** | **Selection**  **(max 4★)** | **Comparability**  **(max 2★)** | **Outcome**  **(max 3★)** | **Total**  **(max 9★)** | **Overall methodological quality** |
| --- | --- | --- | --- | --- | --- |
| Näsström et al., 1985 | ★★★★  Selected from Department of Nephrology; non-transplant renal patients from same source; secure medical treatment groups; baseline radiographs available before treatment | ☆☆  Not reported | ★★★  Outcome assessed without knowledge of diagnosis/treatment; follow-up >2 years; exclusions described (refusal, death, etc.) | 7/9 | High |
| Kansu et al., 2009 | ★★☆☆  Referred from Nephrology Department; medical referral; selection of non-exposed cohort and baseline outcome status not reported | ☆☆  Not reported | ☆☆☆  Outcome assessment, follow-up duration, and completeness not reported | 2/9 | Low |
| Horsley et al., 2009 | ★★☆☆  Patients from dental oncology clinic; retrospective dental record review; non-exposed cohort and baseline outcome status not reported | ☆☆  Not reported | ★☆★  Blinded endodontic assessment; exclusions described; follow-up duration not reported | 4/9 | Moderate |
| Patil; Sinha, 2013 | ★★☆☆  Referred from Nephrology Department; medical referral/diagnosis; non-exposed cohort and baseline outcome status not reported | ☆☆  Not reported | ☆☆☆  Outcome assessment, follow-up duration, and completeness not reported | 2/9 | Low |
| Alsweed et al., 2019 | ★★☆☆  Randomly selected digital radiographs from five hospitals; exposure from digital archives; non-exposed cohort and baseline outcome status not reported | ☆☆  Not reported | ☆☆☆  Outcome assessment, follow-up duration, and completeness not reported | 2/9 | Low |
| Mathew et al., 2019 | ★★★☆  Patients from university clinic; medically fit group from same source; medical history records; baseline outcome status not reported | ☆☆  Not reported | ☆☆☆  Outcome assessment, follow-up duration, and completeness not reported | 3/9 | Low |
| Yilmaz et al., 2019 | ★★★☆  University hospital database; non-exposed cohort from same hemodialysis population; retrospective clinical data; baseline outcome status not reported | ★★  Controlled for age, diabetes, CRP, PTH, Ca×P product, and dialysis duration | ☆☆★  Re-evaluation of 30% of records for reproducibility; outcome assessment and follow-up duration not reported | 6/9 | Moderate |
| Alğan et al., 2025 | ★★★☆  Retrospective selection from Department of Oral Diagnosis; non-exposed cohort from same clinic; medical history records; baseline outcome status not reported | ☆☆  Not reported | ★☆☆  Blinded re-evaluation by two operators; follow-up duration and completeness not reported | 4/9 | Moderate |
| Baghestani et al., 2025 | ★★★☆University dental school cohort; non-exposed group from same community; exposure based on HbA1c records; baseline outcome status not reported | ★★  Matched for age and gender | ★☆★  Masked outcome assessment; all eligible patients included; follow-up duration not reported | 7/9 | High |
| Fairozekhan et al., 2025 | ★★☆☆  Patients from screening clinics; exposure from medical records; non-exposed cohort and baseline outcome status not reported | ★★  Adjusted for age, gender, diabetes, blood pressure, and systemic diseases | ☆☆☆  Outcome assessment, follow-up duration, and completeness not reported | 4/9 | Moderate |

**Supplementary Table S7.** Stepwise thematic synthesis of findings across the included studies (n = 33)

| **Step** | **Findings** |
| --- | --- |
| (i) Familiarization with the data | The initial immersion in the dataset revealed PC as a phenotypically heterogeneous entity, varying from discrete mineralized bodies to diffuse obliteration, frequently conditioned by the resolution limits of the imaging modality employed (thresholds <200 µm). A consistent pattern emerged regarding anatomical distribution, with a marked predilection for molars attributed to pulpal volume and vascularity, suggesting that local biological factors actively modulate the expression of calcification. Furthermore, the data repeatedly highlighted a dichotomy: while strong associations with metabolic (DM) and cardiovascular (CVD) dysregulations support the hypothesis of PC as a marker of ectopic mineralization, the variability in prevalence estimates—often driven by methodologic heterogeneity—underscores a critical tension between biological plausibility and the diagnostic limitations of current observational evidence. |
| (ii) Generating initial codes | Biological codes: Systemic metabolic dysregulation; Vascular micro-calcification; Reparative dentinogenesis; Anatomical vulnerability (molar dominance); Age-dependent accumulation. |
|  | Clinical codes: Diagnostic non-specificity; Screening marker potential; Risk indicator; Systemic susceptibility signal. |
|  | Methodological codes: Imaging bias (2D vs. 3D); Underestimation of prevalence; Heterogeneous outcome definitions. |
| (iii) Searching for themes | Candidate Theme 1: PC as a local expression of systemic metabolic/vascular dysregulation, shared inflammatory and mineral pathways. |
|  | Candidate Theme 2: Anatomical susceptibility and local biological modulation, independent of systemic status. |
|  | Candidate Theme 3: Imaging-driven variability in prevalence. |
|  | Candidate Theme 4: Divergence between etiopathogenic plausibility and clinical utility. |
| (iv) Reviewing themes | Consolidation: Candidate Themes 1 and 4 were merged into a central analytical axis distinguishing "Biological Plausibility" from "Clinical Specificity," preserving the observed tension between mechanistic links and diagnostic limitations. |
|  | Validation: Theme 2 (Anatomy) and Theme 3 (Methodology) were retained as distinct, transversal modulators that explain variability across the dataset. |
|  | Outcome: Establishment of a multifactorial model where PC is biologically meaningful but methodologically contingent. |
| (v) Defining and naming themes | 1. Biological Triggers: Captures the evidence linking PC to CVD, DM, and renal disorders via convergent pathways (inflammation, altered mineral metabolism, vascular compromise), positioning systemic disease as a permissive or amplifying factor. |
|  | 2. Local Modulators: Reflects the consistent predilection for molars and the role of pulp volume, functional loading, and cumulative irritation, indicating that local tissue characteristics modulate the systemic expression. |
|  | 3. Observational Constraints: Encompasses the impact of imaging thresholds (<200 µm) and study design on prevalence estimates, underscoring that the variability in reported associations is largely driven by the resolution of the diagnostic tools employed rather than biological inconsistency. |
| (vi) Producing the report | The analysis supports a conceptual model where PC is a biologically relevant phenomenon arising from the interplay of biological triggers (systemic susceptibility) and local modulators (anatomical substrate). However, the characterization of this association is currently limited by observational constraints, particularly the reliance on low-sensitivity 2D imaging, which obscures the true magnitude of the systemic influence. |

**Supplementary Table S8.** GRADE justification for certainty of evidence assessments.

| **Category** | **GRADE Domain** | **Judgement** | **Justification** |
| --- | --- | --- | --- |
| Overall systemic diseases (Global) | Risk of bias | Downgrade (–1) | Most included studies were observational and showed limitations related to confounding control, outcome assessment, and study design. Residual confounding could not be excluded. |
|  | Inconsistency | No downgrade | Although statistical heterogeneity was substantial (I² = 74.5%), the direction of effect was consistent across studies. Sensitivity analysis suggested that no single study fully explained the association. |
|  | Publication bias | No downgrade | No clear evidence of publication bias was identified by funnel plot inspection or Egger’s test. |
|  | Large magnitude of effect | No upgrade | Although the pooled OR was large (OR = 2.69), upgrading was not applied because of the observational design, residual confounding, and methodological heterogeneity. |
| Diabetes mellitus | Risk of bias | Downgrade (–1) | The evidence was based on observational studies with limited adjustment for relevant local, behavioural, and systemic confounders. |
|  | Inconsistency | No downgrade | Statistical heterogeneity was absent (I² = 0.0%), suggesting consistency across studies. |
|  | Imprecision | Downgrade (–1) | The subgroup included only three studies, limiting confidence in the precision and stability of the estimate. |
|  | Large magnitude of effect | No upgrade | Although the pooled OR was large (OR = 2.30), this was not considered sufficient to upgrade certainty given the small number of studies and potential residual confounding. |
| Atherosclerosis | Risk of bias | Downgrade (–1) | The included studies were observational and may have been affected by residual confounding and differences in radiographic assessment. |
|  | Inconsistency | No downgrade | No statistical heterogeneity was observed (I² = 0.0%), indicating consistent direction and magnitude across studies. |
|  | Large magnitude of effect | No upgrade | Although the association was very large (OR = 4.70), upgrading was not applied because the observational evidence remains vulnerable to bias and confounding. |
| General cardiovascular disease | Risk of bias | Downgrade (–1) | Observational designs and incomplete control of confounding reduced confidence in the estimate. |
|  | Inconsistency | Downgrade (–1) | Moderate heterogeneity was observed (I² = 68.1%), and the pooled estimate did not reach statistical significance. |
|  | Imprecision | Downgrade (–1) | The confidence interval was wide and crossed the null value, indicating substantial uncertainty. |
| Specialised conditions (renal or genetic) | Indirectness | Downgrade (–1) | The included populations were highly specific, limiting applicability to broader systemic disease contexts. |
|  | Imprecision | Downgrade (–1) | The evidence was limited to two studies with small samples and wide confidence intervals. |
| Non-specific and multiple conditions | Risk of bias | Downgrade (–1) | The evidence was observational and included heterogeneous systemic conditions, with potential residual confounding. |
|  | Inconsistency | Downgrade (–1) | Statistical heterogeneity was high (I² = 89.3%), reducing confidence despite a generally consistent direction of effect. |
|  | Large magnitude of effect | No upgrade | Although the pooled OR exceeded 2.0, upgrading was not applied because of substantial heterogeneity, broad clinical grouping, and residual confounding. |
